# Origin, Evolution and Diversity of φ29-like Phages—Review and Bioinformatic Analysis

**DOI:** 10.3390/ijms251910838

**Published:** 2024-10-09

**Authors:** Peter Evseev, Daria Gutnik, Alena Evpak, Anastasia Kasimova, Konstantin Miroshnikov

**Affiliations:** 1 Shemyakin-Ovchinnikov Institute of Bioorganic Chemistry, Russian Academy of Sciences, Miklukho-Maklaya Street 16/10, 117997 Moscow, Russia; 2 Laboratory of Molecular Microbiology, Pirogov Russian National Research Medical University, Ostrovityanova Street 1, 117997 Moscow, Russia; 3 Limnological Institute, Siberian Branch of the Russian Academy of Sciences, Ulan-Batorsakaya Street, 3, 664033 Irkutsk, Russia; 4 Zelinsky Institute of Organic Chemistry, Russian Academy of Sciences, Leninsky Prospekt, 47, 119991 Moscow, Russia

**Keywords:** phage φ29, phage phi29, φ29-like phages, φ29 evolution, phage evolution, phage origin, phage diversity, bacteriophage biodiversity

## Abstract

Phage φ29 and related bacteriophages are currently the smallest known tailed viruses infecting various representatives of both Gram-positive and Gram-negative bacteria. They are characterised by genomic content features and distinctive properties that are unique among known tailed phages; their characteristics include protein primer-driven replication and a packaging process characteristic of this group. Searches conducted using public genomic databases revealed in excess of 2000 entries, including bacteriophages, phage plasmids and sequences identified as being archaeal that share the characteristic features of phage φ29. An analysis of predicted proteins, however, indicated that the metagenomic sequences attributed as archaeal appear to be misclassified and belong to bacteriophages. An analysis of the translated polypeptides of major capsid proteins (MCPs) of φ29-related phages indicated the dissimilarity of MCP sequences to those of almost all other known *Caudoviricetes* groups and a possible distant relationship to MCPs of T7-like (*Autographiviridae*) phages. Sequence searches conducted using HMM revealed the relatedness between the main structural proteins of φ29-like phages and an unusual lactococcal phage, KSY1 (*Chopinvirus KSY1*), whose genome contains two genes of RNA polymerase that are similar to the RNA polymerases of phages of the *Autographiviridae* and *Schitoviridae* (N4-like) families. An analysis of the tail tube proteins of φ29-like phages indicated their dissimilarity of the lower collar protein to tail proteins of all other viral groups, but revealed its possible distant relatedness with proteins of toxin translocation complexes. The combination of the unique features and distinctive origin of φ29-related phages suggests the categorisation of this vast group in a new order or as a new taxon of a higher rank.

## 1. Introduction

Bacteriophage φ29, infecting *Bacillus*, is one of the classical entities of phage biology. First described in the 1960s [1,2,3,4], φ29 has now been the focus of more than 500 research articles, which have featured many aspects of its basic virology, genetics, protein and structural biology. Many features of its molecular organisation and infection cycle have been considered unique to φ29, in contrast to other well-studied phages such as T4, T7 or λ. Subsequently, an accumulation of scientific data on phages sharing some keystone properties has resulted in the establishment of a group of φ29-like phages that infect various Gram-positive and Gram-negative bacteria. Current global changes in the taxonomy of bacteriophages and viruses in general [5,6] require the listing of original genomic and metabolic features that are common to all members of taxa at the level of the subfamily and above.

At present (August 2024), phage φ29 and closely related phages are assigned to the class *Caudoviricetes*, family *Salasmaviridae*, subfamily *Picovirinae*, genus *Salasvirus* (https://ictv.global/taxonomy, accessed on 1 September 2024). Several other families approved by the International Committee on Taxonomy of Viruses (ICTV) families contain phages more distantly related to φ29, infecting Bacillales and Clostridia, which are characterised by varying degrees of similarity of morphogenetic proteins to those of φ29 and protein primer-driven replication. These are *Guelinviridae* (*Clostridium* phage CPV4 [7] and others), *Madridviridae* (e.g., *Streptococcus* phage CP-1 [8]) and *Rountreeviridae* (*Staphylococcus* virus Andhra [9] and others) families. Several actinophages, as were shown previously [10], also appear to be related to φ29, which include the unclassified *Curtobacterium* phages Ayka and those related [10,11], and phages assigned by the ICTV to genera but not included in subfamilies or families; those are genera, namely, *Anjalivirus*, *Badaztecvirus* and *Dybvigvirus* [12]. In addition, several phages infecting Gram-negative bacteria appear also to possess morphogenesis proteins and DNA polymerase similar to φ29 ones, they include *Salmonella* phages assigned to genus *Astrithrvirus*, *Pectobacterium* phage DU-PP-III, and *Acinetobacter* phage Aci01-2-Phanie (short name Phanie) [13].

The diversity of environmental niches and bacterial hosts of φ29-related phages promotes the search of similar phages in metagenomic data, and their unusual features raise questions about the multiformity of this viral group and its origin. The evolutionary analysis of bacteriophages is a cumbersome task. The evolution of viruses is characterised by network character [14] and modular nature [15,16], and viral proteins tend to diverge fast. The last factor can be sometimes overcome by analysing protein folding and structural similarity, which are more conservative than amino acid sequence. This approach is, however, limited by a number of experimentally resolved structures which are much fewer than the available sequences.

φ29-related phages are podoviruses, i.e., phages with short non-contractile tails. The major capsid proteins (MCPs) of these phages, as well as other *Duplodnaviria* viruses and shell proteins of encapsulins, have an HK97-like fold that reflects the common origin of the capsids of tailed viruses and encapsulins [17]. The details of the lineage and the functions of the last common ancestors of phage capsids and encapsulin, though, are still hypothesised. In our previous work [18], the structural models of MCPs and terminases and a phylogenetic analysis based on protein sequences were used to reveal the degree of relationship between 50 phage families classified by ICTV. The clustering that was performed has placed analysed families of φ29-related phages into a clade distinct from other groups, but reliable conclusions about the closest relatives of φ29-related phages can hardly be drawn due to the early divergence of this group, low statistical support (or its absence) and possibly inaccurate models. To the authors’ knowledge, the origin and evolution of short podoviral tails, including those of φ29-like phages, remain even less understood. Previously published results indicate that both the N-terminal domain of the tail knob protein of *Streptococcus* phage C1 (*Rountreeviridae* family) and, possibly, phage φ29 tail knob protein, forming a hexameric ring, have a common protein fold usually found in the neck and tail tube proteins of sipho- and myoviruses. This allows to hypothesise that all three morphological groups of tailed viruses have a common origin [19]. Nevertheless, the authors mention [19] that these “N-terminal domains do not form a continuous β-barrel through tight interactions within a hexamer, as observed in other tube proteins”.

Taxonomy and evolutionary history are tightly connected. The understanding of evolutionary relationships of viral proteins is important to plot a classifying scheme that reflects a “natural” evolutionary relationship between viral taxa. Currently, the classification of viruses is primarily based on the results of genomic analysis. In the current paper, modern genomic and structural prediction tools are used to provide an overview of the typical characteristics of φ29-like phages, to search for representatives in databases including metagenomes, to suggest the evolutionary traits that led to the establishment of this group of bacteriophages, and to reveal evolutionary relationship between the proteins of φ29-related phages and other viruses/cellular organisms. First, the published data on the structure of the genome and virions of φ29-like phages are reviewed for a better understanding of gene functions. Next, a thorough search for sequences similar to phage φ29 protein sequences is performed using different methods. Then, the found genomes are clustered and representative sequences are annotated and studied. Finally, we discuss the results and their implications for phage taxonomy, and propose hypotheses that may help in understanding the evolution of this group of phages.

## 2. Results

### 2.1. Brief Review of Published Studies on Phage φ29 Genomics and Structural Features

#### 2.1.1. General Genomic Features of Phage φ29

The genome of *Bacillus* phage φ29 consists of a linear dsDNA molecule of 19,282 bp (NCBI Accession #EU771092.1) containing short inverted terminal repeats (5′AAAGTA) [20]. Terminal proteins (TPs) are covalently attached at 5′- termini of each strand of the genomic DNA [21]. The φ29 genome contains 27 protein-encoding genes with a coding capacity of 94.8%. There are no tRNA genes in the genome, but it encodes for 174 nt prohead RNA (pRNA, from 147 to 320 bp), which is essential for the packaging of the genome [22,23]. The functions of most proteins have been characterised to a greater or lesser extent.

**Figure 1:**
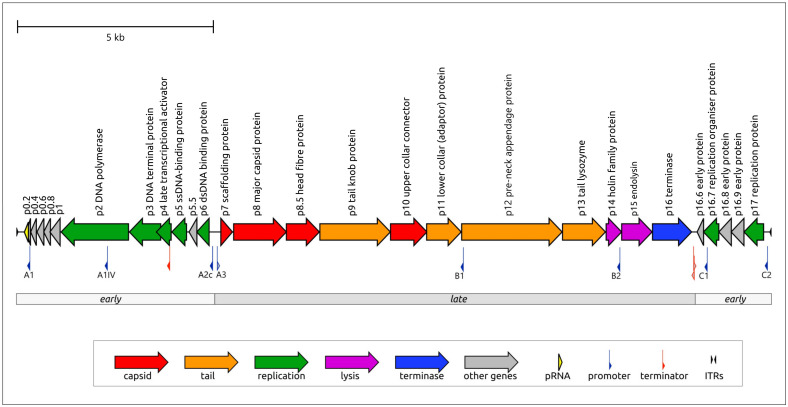
Genetic map of phage φ29 (based on genomic sequence under NCBI Accession #EU771092). Annotations are shown in labels and legends. Arrows indicate the direction of transcription. The scale bar indicates the length of the nucleotide sequence. The names of gene products are shown in accordance with experimentally assigned functions (see text). The grey bars at the bottom of the map indicate the location of the early and late regions.

Like many other tailed phages, the φ29 genome has a distinct modular structure (Figure 1). Three regions, containing blocks of genes responsible for different stages of infection, in order of transcription during the infection cycle, can be distinguished—these are the right and left early regions and a central late region [24,25]. Transcription is regulated by several promoters, including early promoters C1, C2, A1, A2a, A2b and A2c (promoters A2a and A2b are located close to A2c) and late promoters A3, B1 and B2 [26]. Compared with other φ29 promoters, promoters B1 and B2 resulted in minor amounts of RNA synthesis in vivo [27]. Two Rho-independent terminators for the early and late transcripts are positioned between late- and rightward-reading early regions. Another Rho-independent terminator, presumably facilitating the effective production of proteins p5 and p6, is located within gene 4 [28]. The left early region does not appear to contain Rho-independent transcription, meaning that transcription starting from the A1 and A2 promoters continues until the left end of the genome is reached [26]. Promoter A3, which is responsible for the formation of the transcript containing all the late region coding sequences, is regulated by protein p4 (a product of early gene 4); p4 binds upstream from RNA polymerase (RNAP) and the p4-RNAP complex interaction stabilises the polymerase at the promoter [29,30]. Four p4 binding sites are located between the A2c and A3 start sites [31]. The A1IV promoter, located in the coding region of the DNA polymerase, promotes the synthesis of the p1 protein and is weakly expressed in vivo [28,32].

Early regions also contain genes comprising the replication machinery of the phage. Like some other prokaryotic and eukaryotic viruses (adenoviruses [33], tectiviruses [34], *Sulfolobus ellipsoid virus 1* [35]) and linear plasmids [36,37]), phage φ29 features protein-primed replication [38]. The gene of terminal protein p3 is located in the right early region upstream (from the direction of transcription) of the DNA polymerase (DNAP) gene2. The left early region also encodes an unusual dsDNA binding protein, p6, with versatile functions, including participation in the genome organisation of genomic DNA, DNA replication and transcriptional regulation [39,40,41]. The latter function appears in the repression of the early C2 promoter and in the participation in the binding of the p4-RNAP complex upstream of the late promoter A3 and early promoter A2c. The low specificity of DNA-binding functionally relates p6 to prokaryotic histone-like proteins [40]; this relatedness is reflected in the fact that p6 is sometimes called a “viral histone-like protein”. The phage φ29 replication process initially uses p17, which supposedly recruits initiation factors at the replication origins in conditions of low concentrations of other replication proteins and becomes unnecessary after the establishment of the infection process [42]. Protein p17 is encoded by gene17, which is located in the right early region, and is synthesised early at the beginning of infection.

The late region of the φ29 genome comprises a block of structural genes, a lysis module and a DNA encapsidation (packaging) protein (DNA encapsidation ATPase, further referred to as terminase or Ter). The structural module consists of eight genes, seven of which (the exception being the scaffolding protein) are present in the mature virion [43]. Four genes encode the capsid proteins (scaffolding protein p7, major capsid protein p8, head fibre protein p8.5 and upper collar protein p10) and the remaining four genes encode tail structural proteins, including tail protein p9, adaptor p11, pre-neck appendage (tail fibre) protein p12 and lysozyme domain-containing proteins p13, which are localised at the distal tip of the tail knob and participate in the degradation of the cell wall at the penetration stage [44]. The lysis module comprises genes of holin (p14) and endolysin (p15). The last gene of the late region encodes the terminase (p16).

#### 2.1.2. General Characterisation of the φ29 Virion and Proteins

**Figure 2:**
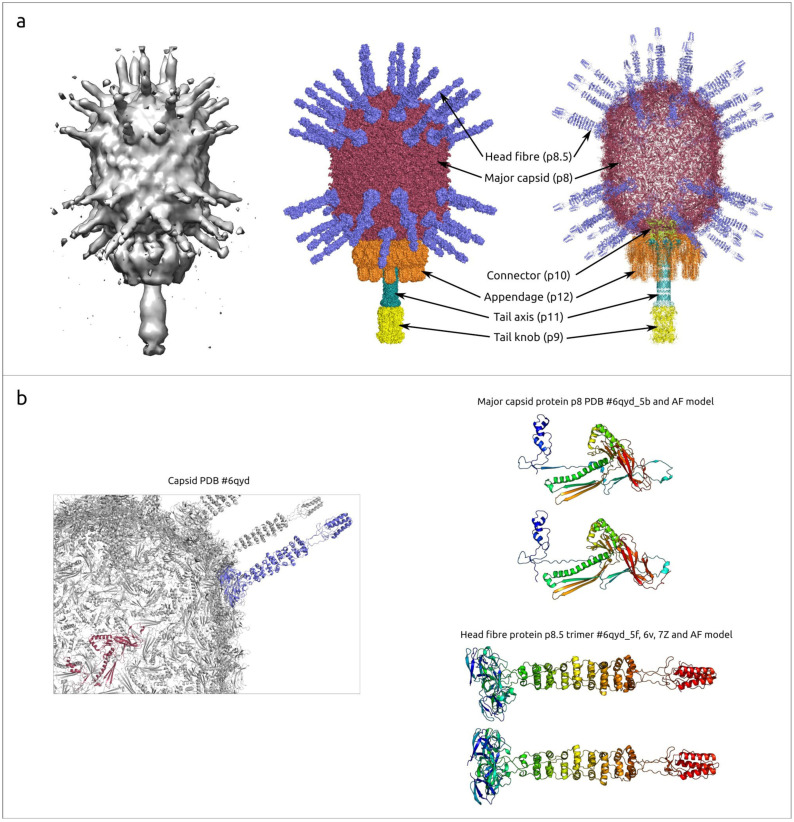

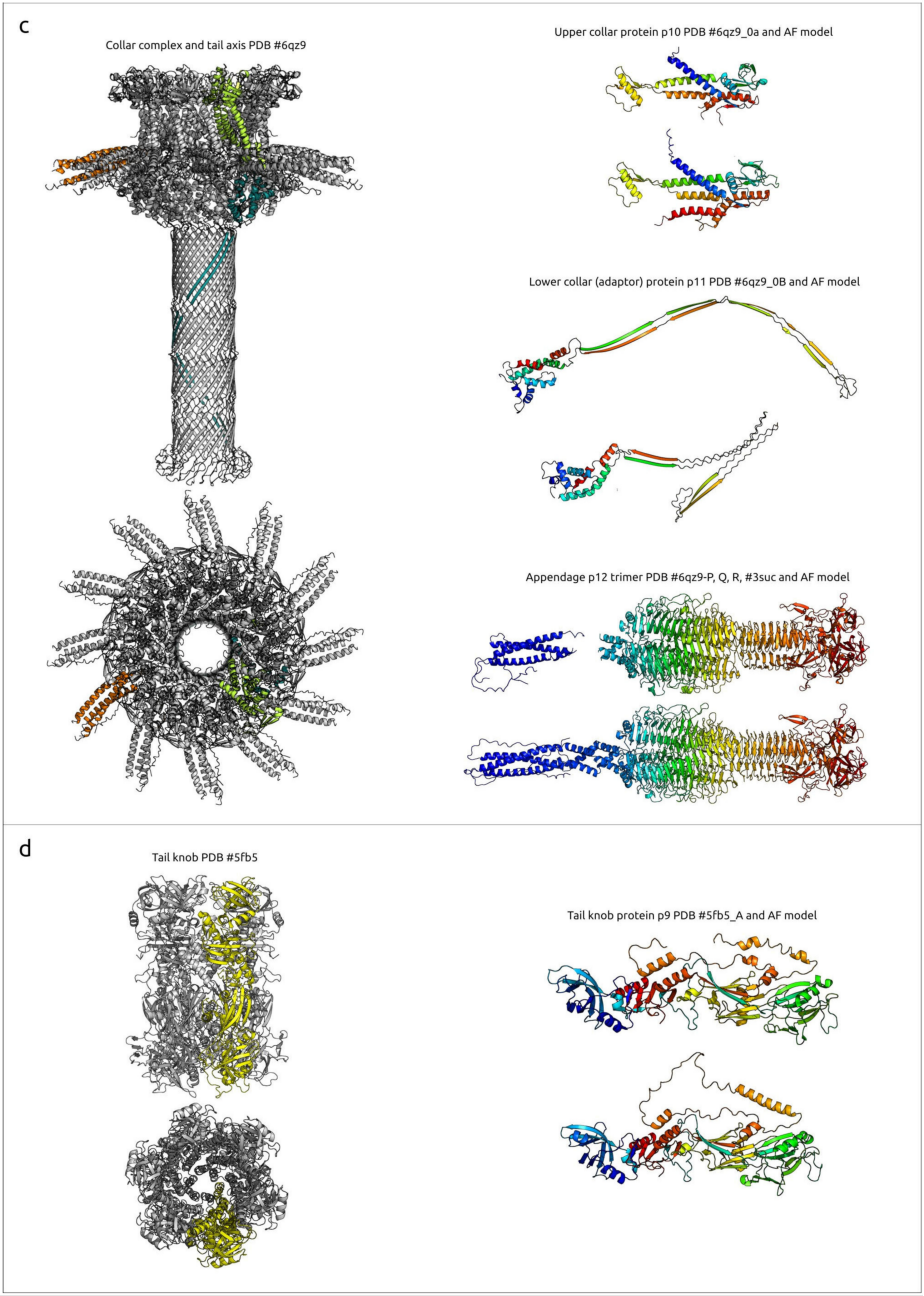
(**a**) A general view of the φ29 phage particle, based on cryoEM data (EMDB entry EMD-1502) [48], and a schematic view of bacteriophage φ29, including surface-rendered and ribbon presentations showing its structural components based on PDB structures 6qyd, 6qz9, 5fb5 and 3gq7 [43,49,50]. (**b**) A ribbon representation of φ29 capsid components, including major capsid proteins and head fibre trimers, and PDB structures and AF models of corresponding proteins based on PDB structure 6qyd. (**c**) A ribbon representation of φ29 tail components, and PDB structures and AF models of corresponding proteins based on PDB structure 6qz9 [43]. (**d**) A ribbon representation of φ29 tail knob, and PDB structures and AF models of corresponding proteins based on PDB structure 5fb5 [49]. Rainbow colouring uses a colour gradient where the N-terminal end is blue and the C-terminus is red.

A mature virion of phage φ29 consists of a prolate head (capsid) and a short non-contractile tail (Figure 2a). The head is about 45×54 nm in size, with a T = 3, Q = 5 symmetry. As with other *Caudoviricetes* viruses, a scaffolding protein is required for correct capsid assembly. Experiments with a temperature-sensitive mutant scaffolding protein, however, showed that the capsid can spontaneously assemble into isometric particles (T = 3) 37 nm in diameter that lack a head–tail connector and also, to a lesser degree, into larger isometric particles (T = 4) 43 nm in diameter [45]. The capsid is decorated with 55 head fibres attached to quasi-3-fold symmetry positions [46]. Each fibre is composed of three monomers of p8.5; the structure of the fibre has a supercoiled triple repeating helix–turn–helix motif (Figure 2b, AF models are provided in Supplementary File S1). These fibres can facilitate the attachment of the phage onto the host–cell wall. The phage connector is located at the portal vertex and is assembled from 12 protomers of upper collar protein (p10) [47] (Figure 2c). The connector contains a disordered region comprising 12 groups of 18-residue loops, N229–N246, which serves as a ‘clamp’, retaining the DNA within the capsid. The tail of phage φ29 consists of a tail tube, a tail knob and 12 tail appendages (Figure 2c,d). The tail tube consists of 12 copies of lower collar protein p10 and the tail knob is built from six copies of tail knob protein p9 [43]. As well as typical phage tail fibres and tail spikes, φ29 appendages consist of three monomers of protein p12; they participate in the recognition of *B. subtilis* cell-wall teichoic acid, which acts as the primary receptor [46].

Phage φ29 and a number of related *Bacillus* phages possess a peculiar packaging apparatus that employs prohead RNA (pRNA) (Supplementary Figure S1) encoded in the 5′ end part of the genome (Figure 1) [51]. According to current knowledge, this is a unique feature inherent in these phages. Five copies of pRNA, together with five copies of terminase p16 and a connector (consisting of 12 copies of p10), constitute the φ29 packaging motor [23,43,52]. pRNA is present during phage DNA packaging but is missing in the mature virion. Full-sized pRNA has 174 bases, but shorter, 120-base pRNA is sufficient for efficient packaging [53]; purified proheads contain 120-base pRNA [54]. pRNA sequences were identified or predicted in other φ29-like phages [55,56]. Using a BLAST search, similar sequences can be found in the genomes of other *Salasmaviridae* phages, including *Bacillus* phages BSP2, BSP4, PZA, Arbo1, vB_BsuP-Goe15 and others. The structure of terminase (protein p16), was determined experimentally. Its structural architecture was found to be similar to that of other dsDNA phages, including the two-domain structure comprising the N-terminal ATPase domain. The C-terminal endonuclease domain in the terminases of non-φ29-like phages cleaves the concatemeric genome [57]. However, since the packaging substrate for φ29 is unit-length DNA with covalently attached terminal proteins of φ29, the endonuclease activity of φ29 terminase C-terminal domain is unnecessary [58]. This domain of φ29 Ter belongs to RNase H superfamily proteins and still retains its DNA binding capabilities [59].

Interestingly, the predicted structures of proteins p0.6 and p0.8 are similar, possibly indicating gene duplication events in the evolutionary history of phage φ29. Tail-associated lysin p13 (TAL, tail lysozyme) is important for penetration into the host–cell. The TAL is, apparently, located at the distal end of the φ29 tail knob. This protein has a two-domain structural architecture; the N-terminal domain can cleave the β(1–4) glycosidic bonds between N-acetylglucosamine and N-acetylmuramic acid residues and the C-terminal domain can cleave cross-links in peptidoglycan layers [48].

Experimentally derived and/or AF-predicted structures of other φ29 proteins are shown in Supplementary Figure S2. Apparently, despite the similarity between the predicted and experimentally determined structure, the orientation of protein domains may be predicted incorrectly.

**Figure 3:**
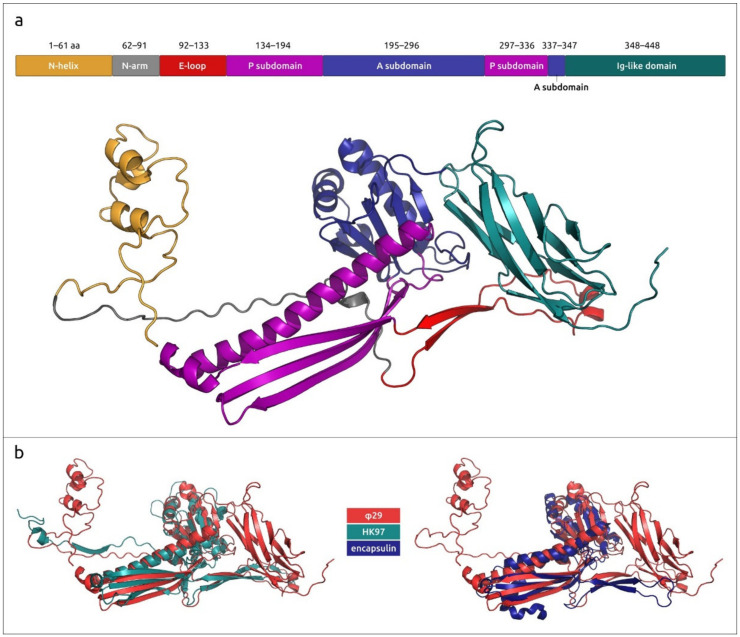
A scheme (**a**) and ribbon diagram showing the domainal structure of phage φ29 MCP (AF model), and the superimposition (**b**) of φ29 MCP (AF model) and PDB databank structures of phage HK97 MCP (PDB #1ohg [60]) and encapsulin from *Thermotoga maritima* (PDB #7kq5 [61]).

Major capsid protein p8, the main component of the φ29 head, is predicted to be a 448 aa protein encoded by gene 8. The experimentally determined structures of the φ29 MCP have unambiguously shown its attribution to HK97 fold proteins. An HK97 fold is characteristic of tailed phages, herpesviruses and encapsulins [17]. The structures of φ29 proteins forming hexons and pentons of capsid are not identical, but very similar (RMSD about 3 Å), as well as being similar to AF models (RMSD about 1–2 Å for both hexons and pentons) (see Figure 3, Supplementary Figure S3). An interesting feature of the φ29 MCP is the presence of an additional all β-strand immunoglobulin-like (Ig-like) domain [43].

Scaffolding protein p7, important for correct head assembly, is missing in the mature virus head. A cryo-EM analysis indicated that the scaffolding protein was organised inside the head as a series of concentric shells; it can bind DNA, and this binding can mediate the structural transition from prohead to mature capsid, liberating scaffolding protein [62]. According to experimental data, scaffolding protein has a helix–loop–helix motif and a disordered tail [43].

The remaining potential proteins encoded in the φ29 genome are non-structural proteins. Most of them take part in processes related to DNA replication and the regulation of protein synthesis. DNA replication is carried out by the phage’s own DNA polymerase (protein) p2, which belongs to the family B of DNA-dependent DNA polymerases and uses the protein primer for the initiation of replication [38,63]. This is a highly processive and accurate DNA polymerase, which implements both polymerisation and 3′–5′ exonuclease activities [64]. To initiate phage DNA replication, p2 interacts with a molecule of terminal protein, encoded by gene3, located in the left early region. During replication, the terminal protein is covalently bound to the phage DNA through a phosphoester bond between Ser232 and 5′-dAMP [65]. Another φ29 protein, p1, is also important for successful replication and was shown to be associated with *Bacillus subtilis* cell division protein FtsZ, colocalising with FtsZ in the medial region of *B. subtilis* [66]. Protein p4, as previously described, participates in the transcriptional regulation, and protein p5 apparently acts as a single-stranded DNA-binding protein, facilitating the DNA-replication-stimulating protein p3 [67]. Protein p6, sometimes referred to as a “histone-like protein”, is structurally completely different to cellular histone proteins. The left early region also encodes several small proteins with an unknown function.

It seems that the proteins of φ29 early genomic regions are responsible mainly for ensuring replication processes. The right early region encodes proteins p16.6, p16.7, p16.8, p16.9 and p17. Protein p16.6 is a small 54 aa protein, whose predicted structure contains a short, double-stranded β-sheet. Protein p16.7 was found to be involved in the organisation of membrane-associated viral DNA replication [26,68]. The structure of p16.7 was determined experimentally (PDB ##1zae [69], 2bnk [69], 2c5r [70]).

Phage φ29 has a comparatively simple two-component lysis machinery; protein p14 acts as a typical phage holin, accumulating in the cytoplasmic membrane and disrupting the host–cell membrane when it reaches a critical concentration [71,72]; protein p15 functions as a muramidase, destroying the cell-wall peptidoglycan [73].

### 2.2. Searches for Similar Proteins Using Sequences and Structures of φ29 Proteins

#### 2.2.1. Major Capsid Protein (p8)

A BLAST search, using sequences of proteins p8 to p11, which compose the virion, indicated a large number of similarities (E-value < 1 × 10^−5^, using the GenBank PHG database) between these proteins and corresponding proteins of phages belonging to the *Salasmaviridae*, *Guelinviridae* and *Madridviridae* families, genera *Anjalivirus*, *Badaztecvirus* and *Dybvigvirus*, and unclassified phages. All these phages have a small genome, which is comparable with φ29 by size, encoding terminal proteins and DNA polymerase and terminase that are related to φ29 counterparts, according to the results of HHpred and BLAST searches.

A search for PDB structures similar to the major capsid protein (p8) using DALI (the DALI search results for p2, p3, p6, p8, p9, p15 and p16 are provided in Supplementary File S2), indicated that several φ29 virion proteins are structurally similar to their functional counterparts in other phages. Search results using experimentally obtained structures, where they have been published, and AF structures, were nearly identical. The AF-predicted structure of φ29 MCP (p8) is most similar to the structures of the MCP of related *Staphylococcus* phage P68 (PDB #6iat [74], DALI Z-score 19.2) and is very similar (Z-score > 6) to that of other phages with podoviral, siphoviral and myoviral morphology.

When using the parts of MCP corresponding to different domains, BLAST and DALI searches found different related proteins. A BLAST search found homologues of Ig-like domain sequence among both *Salasmaviridae* phage capsid proteins and miscellaneous bacterial proteins not related to phages; these were annotated as S-layer homology domain-containing protein, Ig-like proteins, leucine-rich repeat protein, glycoside hydrolase domain-containing protein and others (E-value < 10^−5^, using the nr NCBI database). A DALI search found the closest structure among some bacterial proteins, including β-1,3-glucanase from *Paenibacillus illinoisensis* (PDB #7r3t [75], Z-score 11.9), Ig-like protein from *Leptospira interrogans* (PDB #8gyr [76], Z-score 10.9), S-layer protein Sap from *Bacillus anthracis* (PDB #6hhu [77], Z-score 9.3), etc. and, interestingly, the C-terminal domain of phage tail tube protein from *Escherichia* phage T5 (PDB #5ngj [78], Z-score 11.3). Both BLAST and DALI searches using the domains common for all tailed HK97 proteins (excluding the Ig-like domain) failed to find similar proteins among the proteins belonging to *Bacillus* bacteria. A BLAST search detected homologues of phage φ29 MCP (with the Ig-like domain excluded) among the phage capsid proteins and a DALI search revealed the most similar structures to be phage capsid proteins and encapsulins.

**Figure 4:**
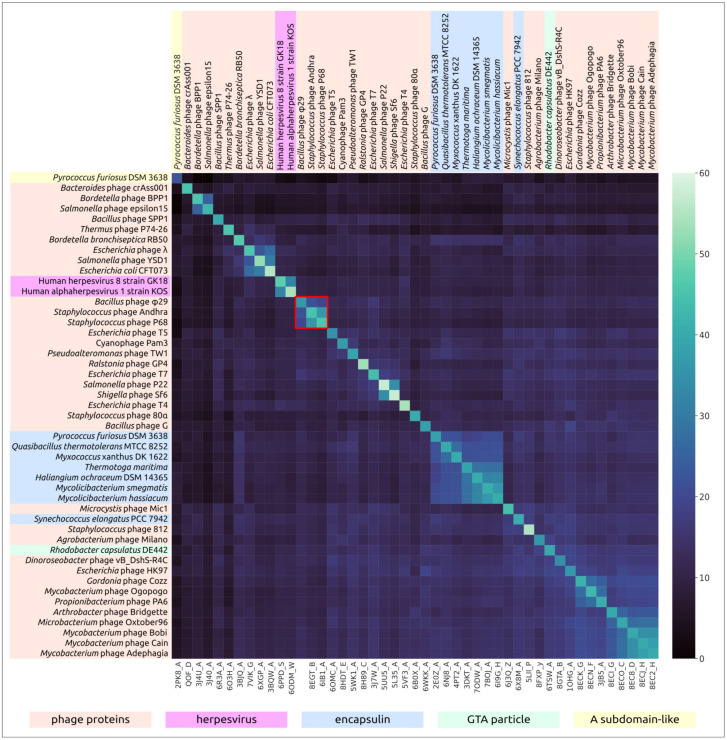
Heatmap based on the pairwise structural similarity of viral capsid proteins and encapsulin major shell proteins, as measured by DALI Z-scores. The cluster of φ29-related phages is outlined in red. The heatmap with numerical values is presented in Supplementary Figure S4.

An HHpred search using the p8 sequence, with the Ig-like domain excluded, revealed an interesting feature of phage φ29 MCP; all related sequences found belonged to small phages that featured protein-primed replication and had terminase and structural proteins related to those of φ29. (Henceforth, these phages will be referred to as “φ29-related phages” or “φ29-related viruses”). Unlike searches using MCP sequences of phages of other classified families of bacteriophages and herpesviruses, the HHpred search found no similar sequences among HK97 viruses (*Heunggongvirae* viruses) that did not belong to φ29-related viruses, even though the DALI search clearly showed structural similarities between the MCPs of phage φ29 and other *Heunggongvirae* viruses. This might have been due to the early divergence of φ29-related viruses. A DALI search indicated the relatedness of φ29 MCP (AF model, with the Ig-like domain excluded) to MCPs of other *Duplodnaviria* viruses and encapsulins, and the most similar structures among the viral capsids, besides other φ29 phages, belonged to *Autographiviridae* capsid proteins (*Klebsiella* phage Kp9 and *Escherichia* phage T7, DALI Z-score 16.2–16.7). The clustering of experimentally determined structures of capsid proteins grouped φ29-like phages in a distinct cluster, separate from other encapsulins, herpesviruses and other phages, including those infecting Gram-positive bacteria (Figure 4, Supplementary Figure S4). Interestingly, this clustering placed almost all encapsulin major shell proteins, except the protein from *Synechococcus elongatus* PCC 7942 (PDB code 6х8m [79]), in a distinct group; the latter one was placed closer to the phage MCPs.

**Figure 5:**
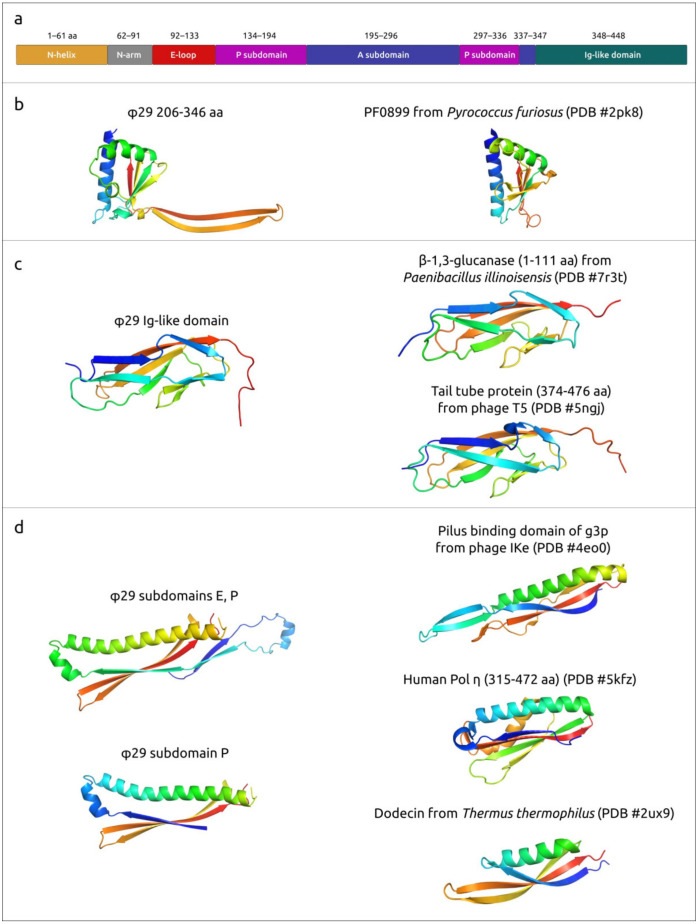
Scheme (**a**) and ribbon diagrams (**b**–**d**) indicating domanial structure similarities between different parts of phage φ29 MCP and similar, experimentally derived non-capsid structures.

A search for more distantly similar structures, apart from HK97 capsid proteins and encapsulins, using separated domains E and P of φ29 MCP, revealed their vague resemblance (Z-score ≥ 4.0) to the pilus-binding domain of an *Escherichia* filamentous phage Ike, eukaryotic DNAP η and bacterial dodecin (Figure 5d). The similarity between dodecin and HK97 capsid proteins was previously noted by Koonin et al. [80]. The A subdomain of φ29 and other HK97 proteins resembles the uncharacterised protein PF0899 from *Pyrococcus furious* (Figure 5b), which was also found previously [81]. As mentioned above, a DALI search also indicated a clear resemblance between the φ29 Ig-like domain and domains of β-1,3-glucanase H from *Paenibacillus illinoisensis* IAM1165 and other carbohydrate metabolism proteins and phage tail tube proteins (Figure 5c). Apparently, the φ29 Ig-like domain was obtained through horizontal gene transfer.

**Figure 6:**
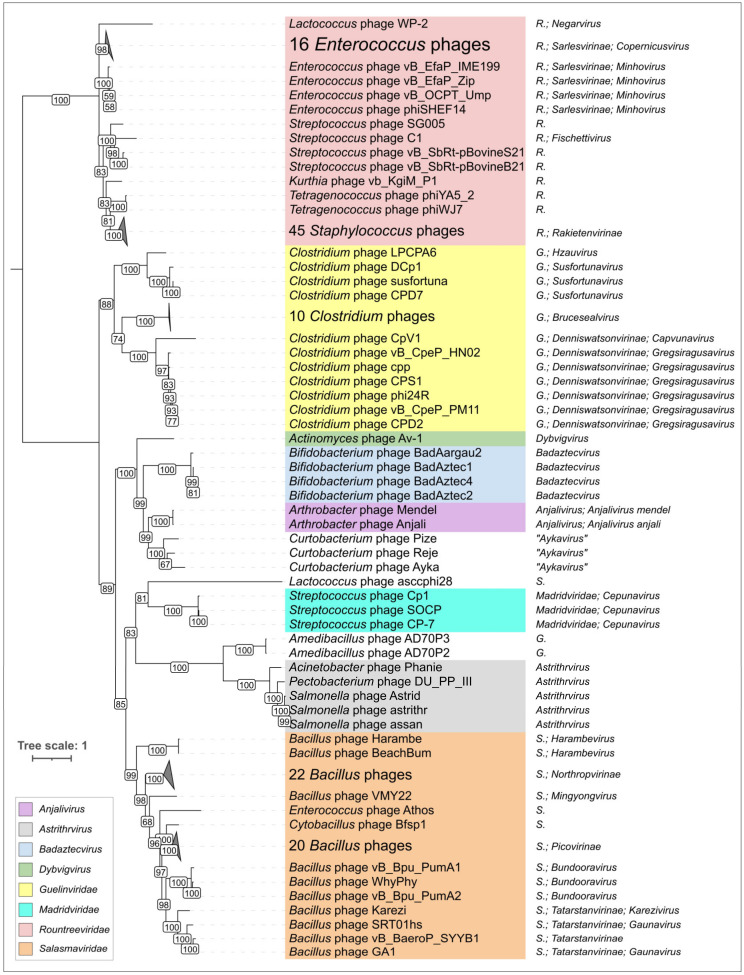
Maximum likelihood phylogenetic trees based on the amino acid sequences of MCP predicted to be encoded in the genomes of isolated φ29-like and related phages. Taxonomy, obtained from the ICTV classification and based on the results of this phylogenetic analysis, is indicated in the legend. Branches with a bootstrap support lower than 50% have been deleted. Some clades have been collapsed, and a tree with all clades expanded is shown in Supplementary Figure S5. Bootstrap values are shown near their branches. The scale bar shows one estimated substitution per site and the tree was rooted to the midpoint.

Since the amino acid sequences of the MCPs of phage φ29 and related phages show no similarity to capsid proteins of other phages, a phylogenetic analysis was conducted using amino acid sequences of MCP encoded in the genomes of phages belonging to currently classified taxa containing φ29-related, as well as recently isolated φ29-related, phages infecting *Curtobacterium* [10,11]. In general, the tree groups φ29-related phages in accordance with the current ICTV taxonomy, but some taxonomy of some phages seems to be needed for clarification (*Lactococcus* phage asccphi28, *Curtobacterium* and *Amedibacillus* phages), (see Figure 6, Supplementary Figure S5). Interestingly, the tree places phages infecting Gram-negative bacteria and Actinobacteria in two single clades, but the *Enterococcus* and *Streptococcus* phages are polyphyletic groups. The distinct, although partially similar, topologies of the phage and bacterial trees (Supplementary Figures S5 and S6), as well as the polyphyletic nature of φ29-related phages in terms of their host taxonomy, indicate that the evolution of φ29-related phages was both coevolutionary and may have involved host switching. A phylogenetic analysis using MCP sequences conducted in a previous study [10] showed the close relatedness of φ29-related phages infecting Actinobacteria and the unclassified, unpublished phage *Rhizobium* phage RHph_N3_8, but an analysis of predicted proteins indicated that this phage appears likely to infect an *Actinobacteria bacterium*.

#### 2.2.2. Head Fibre Protein (p8.5)

A DALI search revealed that the N-terminal part of head fibre protein p8.5 is reminiscent of minor capsid proteins of several other phages of different morphology, e.g., phage YSD1 (PDB #6xgq [82], Z-score 8.1). The similarity of the N-terminal part of p8.5 to the N-terminal parts of minor capsid proteins of other phages was also indicated by HHpred (e.g., siphovirus *Pseudoalteromonas* phage TW1 HHpred probability 97.45% [83]). Also, the HHpred found similarity between the 83 aa C-terminal fragment of p8.5 and a fragment of the head fibre protein gp21 of *Bacteroides* phage crAss001 [84] (HHpred probability 99.3%). A DALI search did not detect significant structural similarities between the C-terminal part of p8.5 and other proteins (the Z-scores for all matches found, except φ29, were 2.9 or lower).

#### 2.2.3. Tail Knob Protein (p9), Upper Collar Protein (p10) and Lower Collar Protein (p11)

The upper collar protein p10 is structurally similar to portal proteins from many other phages, including those distantly related (*Bacillus* phage GA1, PDB #7pv2 [85], Z-score 22.2; *Escherichia* phage T7, PDB #6tjp [86], Z-score 9.8; *Escherichia* phage λ #8k38 [87], Z-score 7.8; *Escherichia* phage T4 #3ja7 [88], Z-score 6.0) and, remarkably, the counterpart from the GTA particle *Rhodobacter capsulatus* (PDB #6toa [89], Z-score 7.4). The upper collar protein has a structure similar to that of other phages of different morphotypes, including podoviruses, siphoviruses and myoviruses. Interestingly, the structure of p10 has similarities with 30S ribosomal protein (r-protein) S15 (PDB #5lmv [90], Z-score 6.2, and other PDB entries), as 30S r-protein S15 is the primary binding protein that orchestrates the assembly of ribosomal proteins S6, S11, S18 and S21 with the central domain of 16S ribosomal RNA to form the 30S subunit platform [91].

**Figure 7:**
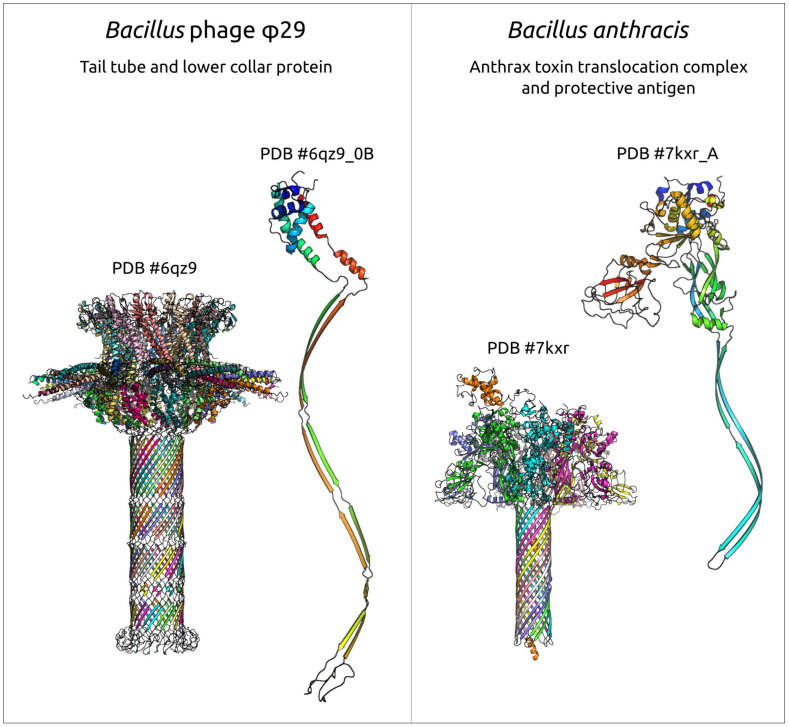
The tail tube of phage φ29 and lower collar protein p11, and anthrax toxin translocation complex and protective antigen protein from *Bacillus anthracis*. In ternary complexes, different chains have a different colour. Separate monomers are shown using rainbow colouring, with a colour gradient where the N-terminal end is blue and the C-terminus is red.

The lower collar protein p11 has no structural similarity to proteins from non-φ29-like phages but has a fairly obvious relationship to analogues from related phages. The lower collar protein shows a similarity to the counterpart of *Staphylococcus* phage Andhra (PDB #8egr [9], Z-score 11.5) and *Staphylococcus* phage P68 (PDB #6iac [74], Z-score 10.3). It is noteworthy that the lower collar protein p11 has a detectable level of structural similarity with the protective antigen of the anthrax toxin translocation complex of *Bacillus anthracis* (PDB #7kxr [92], DALI Z-score 4.4; see Figure 7) and with the tube-forming proteins of toxin translocase complexes of other Gram-positive bacteria, e.g., *Clostridium difficile* (PDB #6uwr [93], DALI Z-score 4.2). The overall architecture of these toxin translocation complex tubes and part of the phage tail formed by the φ29 protein p11 is also similar, but differs in the number of monomers that make up the tube. A structural alignment using extended β-sheet domains of the proteins that make up the tube and lower collar protein φ29 and similar structures from translocase complexes yields RMSD of 5.7 Å and 6.6 Å for 7kxr and 6uwr, respectively.

The tail knob protein p9 is structurally close to its counterpart from *Staphylococcus* phage Andhra (PDB #8egs [9], Z-score 19.6) but, surprisingly, its N-fragment has a structural similarity to bovine cationic trypsin (PDB code 7al8 [94], Z-score 6.3). Another interesting case of structural similarity of apparently evolutionarily distant proteins is that between the N-terminal domain of φ29 tail knob protein p9 and the stopper protein of the GTA particle of *Rhodobacter capsulatus* (PDB #6te9 [89], Z-score 4.4). The N-terminal domain of phage φ29 has the same fold as major tube proteins of myoviruses and siphoviruses [19]. A DALI search conducted using the separated structure of the N-fragment only indicated (PDB #5fb5 chain A) its strong resemblance to tail knob proteins of φ29-related phages (Z-score ≥10.2) and a significantly weaker resemblance to the tail tip protein of phage λ (PDB #8xcg [95], Z-score 5.8), flavoredoxin and other proteins (Z-score 5.4 and lower).

#### 2.2.4. Appendage Protein (p12)

Protein p12, which makes up φ29 appendages, has a strong structural similarity to its counterpart of related *Staphylococcus* phage Andhra (PDB #8egr [9], Z-score 20.5) and tail spikes of other phages (podovirus *Salmonella* phage Sf6, PDB #2vbm [96], Z-score 33.5; myovirus *Escherichia* phage CBA120, PDB #6w4q [97], z-score 31.6). Interestingly, an HHpred search pointed to tail fibres of a t7-like marine podophage carin-1 [98] as to the closest non-φ29-like phage sequence (HHpred probability 100%, e-value 5.7 × 10^−54^). the appendage protein p12 has a noticeable sequence similarity with other *salasmaviridae* phages and many phages belonging to viral groups of very different genome architecture and lifestyle, including the *spbetavirus* genus (genome size of about 130 kb), the *autographiviridae* and *kyanoviridae* families, and other taxa. Also, a blast search found close homologs of p12 in among multiple bacillales proteins (an e-value of about 10^−51^ and lower). This should be a consequence of the evolutionary history of phage receptor-binding proteins (RBPs) accompanied by lateral transfers of RBP domains [99].

#### 2.2.5. DNA Polymerase (p2), Terminal Protein (p3) and “Histone-like Protein” (p6)

There are several experimentally determined structures of φ29 DNA polymerase including PDB #1xhz [100], 2pzs [101] and others. The structures are very similar to each other and to the AF-predicted structure (the superimposition of 1xhz and AF structure onto 2pzs gives RMSD 0.646 Å and 0.880 Å, respectively). However, the search results are not identical, even though the searches give similar Z-score and RMSD values and lists of found PDB entries. Using the 1xhz structure, DALI points to Human DNA polymerase α (Pol α) catalytic subunit (PDB #5iud [102], Z-score 14.2) as the most similar structure, as well as finding numerous eukaryotic polymerases, and archaeal, bacterial and viral B-family polymerases.

**Figure 8:**
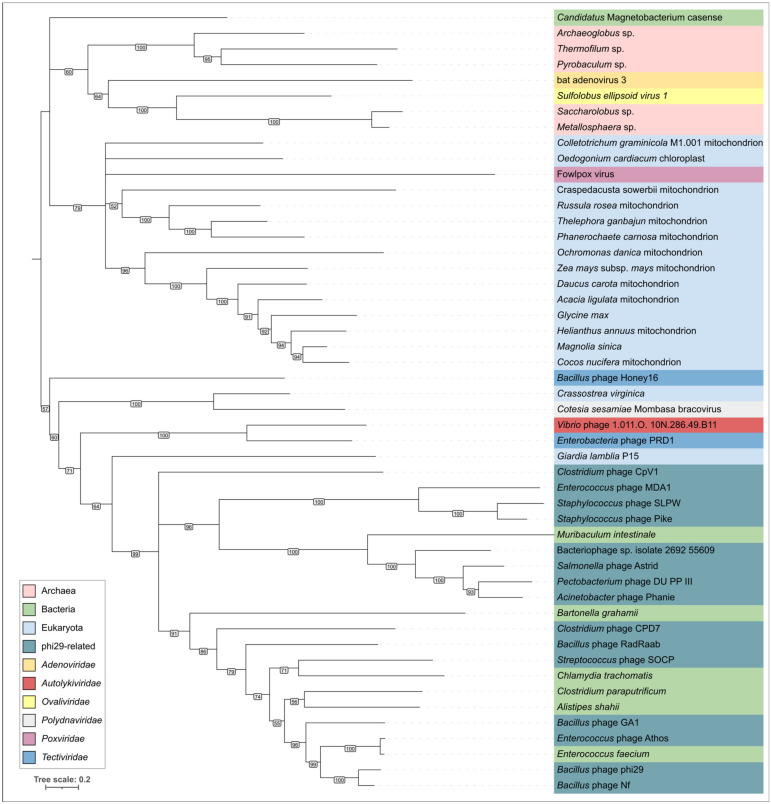
Maximum likelihood phylogenetic trees based on amino acid sequences of DNA polymerases related to φ29 DNAP, according to the results of BLAST and HHpred searches. Taxonomy is indicated in the legend. Branches with a bootstrap support lower than 50% have been deleted. Bootstrap values are shown near their branches. The scale bar shows 0.2 estimated substitutions per site and the tree was rooted to the midpoint.

An HHpred search indicated the resemblance of φ29 DNA polymerase to the DNAP of a φ29-like phage *Streptococcus* Cp-1 (HHpred probability 100%, E-value 4.9 × 10^−54^) and viruses, not belonging to tailed phages, including *Enterobacteria* phage PRD1 (HHpred probability 100%, E-value 6.2 × 10^−42^), *Salterprovirus* His1 (HHpred probability 100%, E-value 7 × 10^−41^), adenoviruses, among archaeal, bacterial and eukaryotic polymerases. BLAST searches showed the clear resemblance of φ29 DNA polymerase to counterparts of phages belonging to the family *Salasmaviridae*, family *Guelinviridae*, family *Madridviridae*, family *Rountreeviridae*, genera *Anjalivirus*, *Astrithrvirus*, *Badaztecvirus* and *Dybvigvirus*, unclassified small-tailed phages and, interestingly, phages of the *Tectiviridae* family infecting both Gram-positive and Gram-negative bacteria. Tectiviruses show a distinct homology between their DNA polymerase and the DNAP of phage φ29 (E-value of 10^−11^–10^−7^, using the GenBank PHG database). In addition, homologous sequences were found in genomes identified as being Firmicutes plasmids, containing phage structural genes and apparently belonging to *Tectiviridae* or *Salasmaviridae*-related phage groups. Phylogenetic analysis using DNAP sequences placed φ29-like phages in a distinct clade, together with bacteria, not far from *Autolykiviridae* and *Tectiviridae* phages and distant from archaea, archaeal and eukaryotic viruses and mitochondria (Figure 8).

A BLAST search using a sequence of the terminal protein (TP) p3 found homologous sequences belonging to φ29-like phages infecting Bacillaceae and various mainly metagenomic bacterial sequences. An HHpred search found meaningful matches only between the TPs of phage φ29 and closely related (family *Salasmaviridae*) *Bacillus* phage Nf (HHpred probability 100%). A DALI search using the p3 PDB structure 2ex3 (chain J) did not find similar structures among experimentally determined phage proteins but indicated some level of similarity of the C-terminal coiled-coil-like domain of p3 to various structures, including an amino-terminal coiled-coil domain of the ebolavirus polymerase cofactor VP35. VP35 binds blunt ends of dsRNA ebolavirus genome and is important for a number of crucial processes including viral replication, being a part of viral RNA-dependent RNA polymerase complex [103]. N-terminal coiled-coil is essential for RNA polymerase function [104], but the dsRNA-binding function is associated with the C-terminal domain [105].

The φ29 “histone-like” protein p6, according to the BLAST search results, has close homologs among the proteins encoded in genomes of *Salasmaviridae* phages and *Enterococcus faecium* strains. Neither HHpred nor DALI indicated strong matches to known protein sequences and determined structures.

#### 2.2.6. Terminase (p16)

Like the large subunit of terminase found in unφ29-like phages, the φ29 terminase has a two-domain structure contained the N-terminal ATPase domain (approx. 1–230 aa) and the C-terminal domain. A BLAST search found viral homologues of φ29 terminase in phages belonging to the *Salasmaviridae* family, *Guelinviridae* family and *Madridviridae* family, genera *Anjalivirus*, *Astrithrvirus*, *Badaztecvirus* and *Dybvigvirus*, and unclassified phages, but not amongst the unφ29-like phages. The use of HHpred allowed us to identify remote homology between the N-terminal part of φ29 terminase, and ATPase domains of various tailed phages with different morphology and herpesviruses (a HHpred probability of 97.5% or higher and an E-value of 0.0069–2.3 × 10^−9^), and the most similar terminases were found in phages *Bacillus* SPP1 and *Shigella* Sf6 (a P22-like podophage of the *Lederbergvirus* genus). At the same time, HHpred failed to detect any significant similarity between the C-terminal domain of φ29 Ter and the endonuclease domains of terminases that do not belong to φ29-like phages. A DALI search using the ATPase domain of φ29 terminase (extracted from PDB structure #7jqq [52]) found similarities between the φ29 N-terminal ATPase domain terminase and the terminases of other dsDNA viruses (including phages of different taxa and morphotypes and herpesviruses) (DALI Z-score 8.3 and higher). Both HHpred and DALI show a similarity of the ATPase domain of φ29 terminase and of DEAD/DEAH-box RNA helicases and other proteins characterised by ASCE fold, which has been noted previously [106].

A DALI search using the C-terminal domain of φ29 terminase (PDB structure # 7cnb [107]) found strong similarity to φ29-like phage terminases (DALI Z-score 8.9 and higher) and slight resemblance to only one non-φ29-like phage terminase, *Escherichia* phage P22 (DALI Z-score 3.8, PDB #4dkw [108]). However, when using another experimentally derived structure of the C-terminal domain extracted from structure PDB #7jqq [52], DALI did not detect its resemblance to non-φ29-like phage terminases, possibly due to the relatively low degree of structural similarity. A DALI search using the C-terminal domain of φ29 terminase AF model indicated some similarity to the endonuclease domains of phages HK97, SPP1 and P22 (DALI Z-score 3.0–4.1).

#### 2.2.7. Lysozyme (Endolysin) (p15)

φ29 endolysin was predicted to have a two-domain structure, typical for many phage endolysins [109], where an N-terminal globular domain is an enzymatically active domain (EAD) linked to a C-terminal domain that may be a cell-wall-binding domain (CBD) (Supplementary Figure S2). The functional roles of φ29 endolysin domains are supported by the results of the BLAST, HHpred and DALI analyses. HHpred indicated the relatedness of the EAD to muramidase domains of a prophage endolysins from *Acinetobacter baumannii* [110] (HHpred probability 99.49%) and different phages. A DALI search indicated a high similarity of the φ29 endolysin EAD to the muramidase domain of the phage origin of SpmX from *Asticaccaulis excentricus* (DALI Z-score 23.0, PDB #6h9d [111]). In turn, the CBD resembles several sugar-binding proteins (HHpred probability 87.14–95.02%, DALI Z-score up to 9.3). Interestingly, a BLAST search using both the EAD and CBD sequences found highly homologous (E-value < 10^−57^ using the GB PHG database) sequences among endolysins of a dozen (but not all) closely related φ29-like phages of the *Salasmaviridae* family which were considerably homologous, belonging to different evolutionary distant phages (E-value < 10^−9^). Also, BLAST searches indicated that the φ29 endolysin has a high homology to cell-wall hydrolases from different bacilli (E-value 0.0069–2.3 × 10^−9^). This corresponds to previous findings that φ29 endolysin has a noticeable level of homology to lysozymes from such evolutionary distant phages as *Escherichia* phage T4 and *Salmonella* phage P22 [112]. The obtained results indicate a complex evolutionary history of endolysins, including horizontal transfer events, which may complicate the reconstruction of evolutionary history based on vertical inheritance.

### 2.3. Search for φ29-Related Viruses

A search for φ29-related viruses was performed using BLAST (tblastn algorithm), amino acid sequences of φ29 MCP and terminase and three GenBank databases: nt, Genome and GB PHG. In addition, the proteins predicted to be encoded in all the GB PHG genomes were checked for relatedness to φ29 MCPs using HHblits. Genomes with an arbitrary chosen cutoff of a genome length of more than 7500 bp were taken for testing and analysis. Combined searches produced 2115 genomic sequences that contained both MCP and Ter genes (Supplementary Table S1). Interestingly, a BLAST search using MCP sequences and the NCBI nt database found phages classified as belonging to the families *Salasmaviridae*, *Guelinviridae* and *Madridviridae*, and genera *Anjalivirus*, *Astrithrvirus*, *Badaztecvirus* and *Dybvigvirus*, while an HHblits search using MCP sequences and the GB PHG database added representatives of the *Rountreeviridae* family and *Lactococcus* phage KSY1 (*Chopinvirus KSY1*) to the previous results. The latter phage has a much larger genome size, evolutionarily distant DNA polymerase and terminase, and will be discussed separately (see Section 2.7). A phylogenetic analysis of MCPs of φ29-related viruses indicated the separate position of *Rountreeviridae* phages from *Salasmaviridae* and other φ29-related phages (Figure 5, Supplementary Figure S5), which explains why they were not found using a BLAST search only.

Surprisingly, 48 NCBI sequences were identified as archaeal metagenomic sequences from the gastrointestinal tract, including 45 Euryarchaeota, 1 *Candidatus* Thermoplasmatota and 2 unclassified Archaea obtained from horse faeces from the endurance horse Fontainebleau. Twenty-four of the sequences were defined as being *Enterococcus faecalis* plasmid and one sequence was identified as a *Clostridium beijerinckii* plasmid. The remaining 2042 sequences were listed as viruses, but only 169 of them were attributed to specific viral taxa at the family level and below, (the same taxa specified as being related in the previous section, in the families *Guelinviridae*, *Madridviridae*, *Rountreeviridae* and *Salasmaviridae*, and in the genera *Anjalivirus*, *Astrithrvirus*, *Badaztecvirus* and *Dybvigvirus).* The length of the sequences found ranged from 7914 bp (MAG, Euryarchaeota archaeon isolate USDE_maxbin.043 k141_1210644) to 34,630 bp (*Enterococcus faecalis* strain BE54 plasmid pBE54_1); GC content ranged from 22.8% (MAG, *Caudoviricetes* sp. isolate ctjXJ9) to 65.9% (MAG, *Caudoviricetes* sp. isolate ctfy85). According to GenBank attributes, isolated viruses infected the Gram-negative bacteria of genera *Acinetobacter*, *Pectobacterium*, *Rhizobium* (questionable) and *Salmonella*, and Gram-positive bacteria of genera *Actinomyces*, *Amedibacillus*, *Arthrobacter*, *Bacillus*, *Bifidobacterium*, *Clostridium*, *Curtobacterium*, *Cytobacillus*, *Eggerthella*, *Enterococcus*, *Glutamicibacter*, *Hungatella*, *Kurthia*, *Lactococcus*, *Microbacterium*, *Staphylococcus*, *Streptococcus*, *Tetragenococcus* and *Weissella*.

### 2.4. Clustering of φ29-Related Viruses

**Figure 9:**
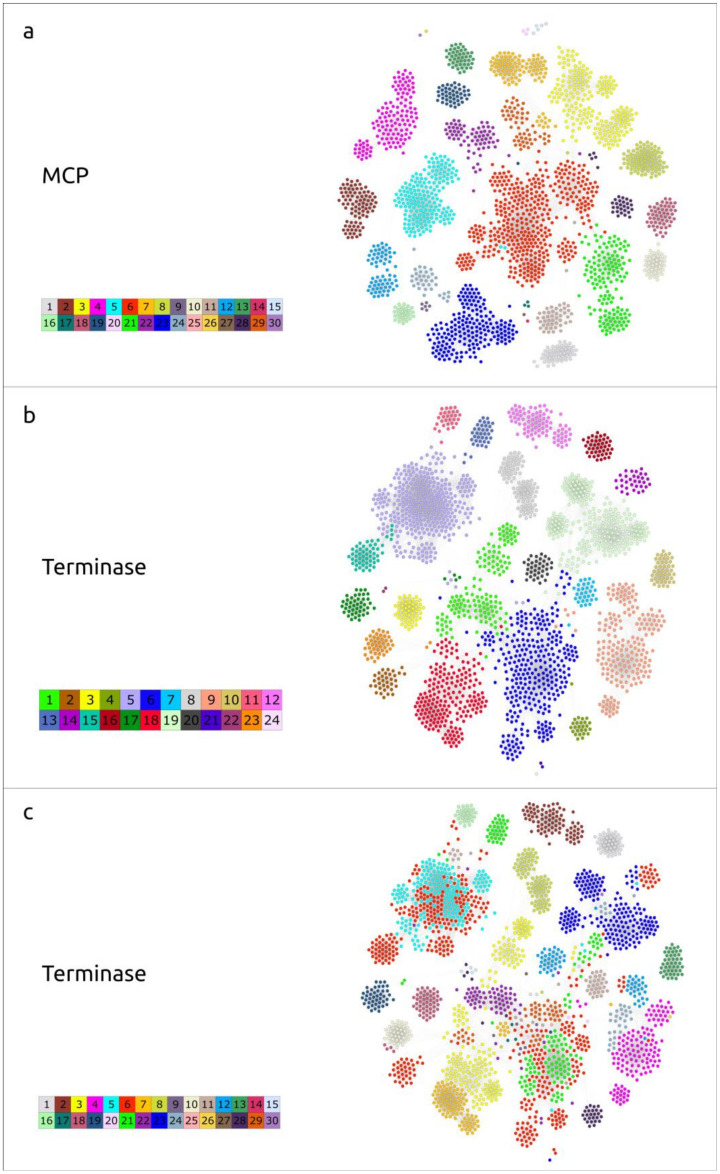
Networks showing the distribution of 2115 MCP sequences generated using the Modularity clustering algorithm in Gephi. Each node corresponds to a distinct sequence of the capsid protein (left panel) and terminase (right panel). All nodes are coloured in different colours and numbered according to their membership in clusters and legends (Supplementary Table S1). (**a**,**c**) Nodes are coloured according to the results of MCP clustering; (**b**) nodes are coloured according to the results of terminase clustering.

To cluster the 2115 sequences found, two separate networks were constructed, using Ter and MCP sequence alignments based on identity scores from DIAMOND BLASTP. Using the Modularity clustering algorithm in Gephi, 30 clusters among MCP sequences, and 24 clusters among Ter sequences were identified (Figure 9, Supplementary Table S1).

Despite the difference in the number of clusters in these two groups, the general pattern of MCP and Ter distribution was similar; most members of classified phage groups were grouped together into the same cluster, for both the major capsid protein and terminase. For example, 56 (out of 60 in total) of the phages belonging to the *Salasmaviridae* family, according to NCBI Taxonomy attributes, were placed in MCP cluster 1, together with two sequences classified as *Enterococcus faecium* and 12 unclassified bacteriophages. In the case of the Ter clustering, the situation was as follows: the same 56 *Salasmaviridae* phages and two sequences from *Enterococcus faecium* were arranged together in the same cluster, which also contained three metagenomic sequences attributed as *Methanobacteriaceae* and 85 unclassified bacteriophages. The clustering of the remaining representatives of the *Salasmaviridae* phages, with both MCP and Ter, was similar: those of *Lactococcus* phage asccphi28 were placed in distinct clusters and three others were arranged together in other distinct clusters.

A noticeable difference can be observed when analysing the clustering of the *Guelinviridae* phages. According to the results of the MCP clustering, the representatives of the family *Guelinviridae* were arranged into three distinct clusters: *Clostridium* (twenty-three phages placed in cluster 29), *Amedibacillus* (two phages placed in cluster 24) and *Eggerthella* phage (one phage in cluster 6). In the case of terminase, *Clostridium* phages were split almost equally (by 11 and 12 representatives) between two clusters: 6 and 18, respectively. Cluster 6 also included the *Eggerthella* phage, with *Amedibacillus* phages allocated to cluster 9. Cluster 18, according to the Ter grouping, was a mixture of representatives of different taxa; it included species belonging to *Guelinviridae*, *Anjalivirus* and *Dybvigvirus*.

The smaller number of clusters for the terminase sequences could, presumably, be explained by its more conserved nature, compared with the major capsid protein. Differences in clusterisation may also suggest recombination events in evolutionary history leading to the emergence of mosaic genomes. Among the Ter clusters, there was only one ‘singleton cluster’—cluster 24—compared with five singleton clusters in the MCPs group. This CANXRI000000000 sequence fell far out of the coherent picture of the network constituted by terminase. Given that MCP is a hallmark protein characteristic of most known viruses, further genomic analyses were conducted based on the results of MCP clustering.

### 2.5. Genome Organisation of φ29-Related Viruses: Main Structural Proteins

**Figure 10:**
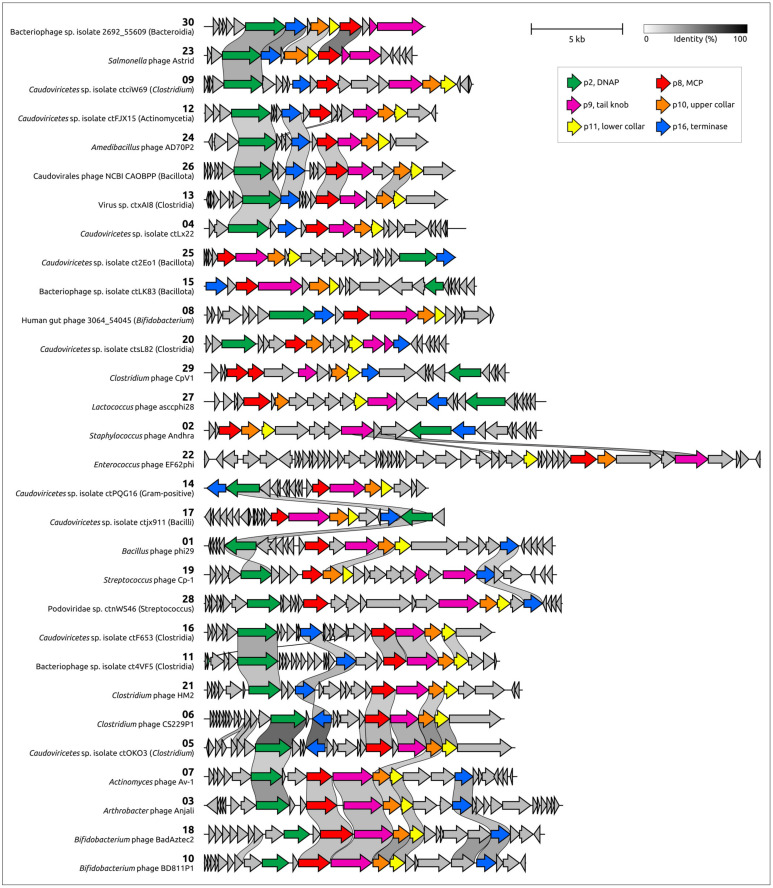
Genetic map of φ29-related viruses. Annotations are shown in labels. Arrows indicate the direction of transcription. The scale bar indicates the level of identity between genes according to a black white gradient, the legend for which is shown in the upper-right of the figure. The number of clusters and the name of sequences are shown on the left. Genes encoding proteins similar to φ29 proteins p2, p8–p11 and p16 are coloured according to the legend.

Genome organisation within the MCP clusters was similar, although some rearrangements can be observed. The general features of arbitrarily chosen representatives from each cluster are shown in Table 1. Only 14 clusters contained isolated phages with a known host. Putative hosts for other phages and possible viruses attributed as Archaea were suggested based on the similarity of predicted proteins, primarily cell-wall-degrading enzymes, to proteins encoded in cellular and viral genomes found in NCBI databases. Apparently, putatively archaeal sequences were misclassified, and belong to bacteriophages. HHblits/HHpred and DALI searches using AF models of predicted proteins indicated that all representative sequences of φ29-related viruses contain proteins related to φ29 proteins p8 (major capsid protein), p9 (tail knob protein), p10 (upper collar protein), p11 (lower collar protein), p2 (DNA polymerase) and p16 (terminase) (Figure 10). These searches indicated that the most similar sequences show either similarity to proteins of φ29-related viruses only, or they are more similar to their counterparts from φ29-related viruses than to proteins from other viruses or cellular organisms. It appears that the ‘main’ morphogenetic proteins (p8–p11), as well as DNAP and Ter of φ29-related viruses, share a common origin and are a ‘hallmark’ of the whole group. The BLAST and HHblits/HHpred searches showed that cell-wall-degrading enzymes or protein domains of endolysins and receptor-binding proteins are often more similar to their counterparts from phages that are evolutionarily distant from φ29-related viruses, or from bacteria. This indicates horizontal transfers of cell-wall-degrading enzymes between phages, with the possible involvement of their hosts. It is often not easy to identify the protein cell-wall-degrading domain to which they belong; genomes of φ29-related phages can encode different proteins, including tail tip, tail or head spike proteins and endolysins, that possess peptidoglycan-degrading activity. Interestingly, some sequences contain characteristic plasmid genes, including all three components of a typical plasmid segregation system (a ParA-type ATPase gene, a gene of a DNA-binding adapter protein with the RHH fold, and a region of DNA with repeats, to which the adapter protein probably binds) as well as a toxin–antitoxin system, suggesting that such sequences belong to phage plasmids, which is in agreement with NCBI GenBank annotations (i.e., cluster 22, *Enterococcus faecalis* BE32 plasmid pBE32_2) [113]. Most φ29-related phages, including all classified phages with genome sequences published by early 2024, do not appear to contain tRNA genes, but some sequences belonging to clusters 04–06, 08, 11, and 21 have been predicted to encode tRNAs.

**Figure 11:**
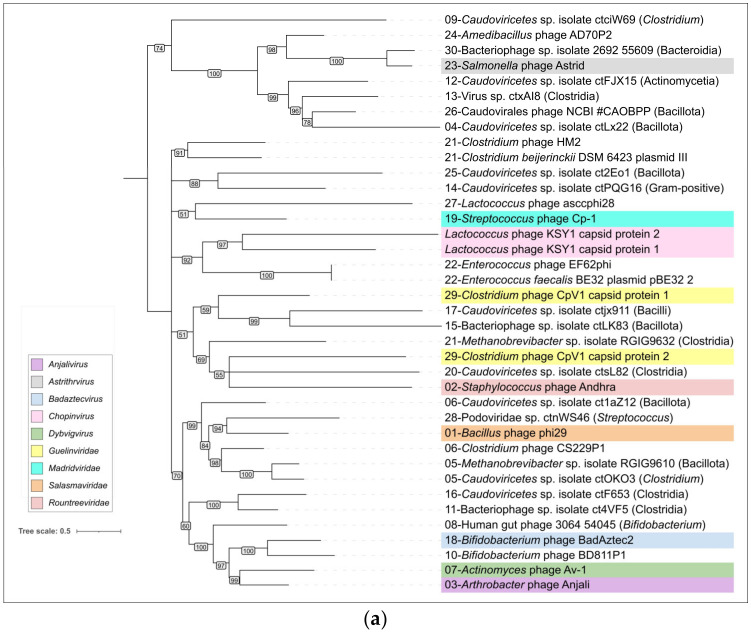

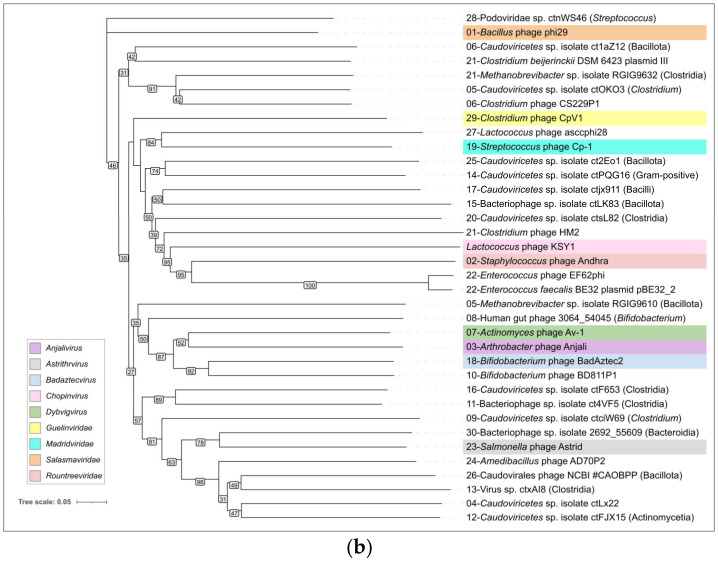
(**a**) Maximum likelihood phylogenetic trees based on amino acid sequences of MCP, predicted in sequences representing clusters of φ29-like and related phages. The taxonomy of isolated phages is indicated in legends. Branches with a bootstrap support lower than 50% have been deleted. Bootstrap values are shown near their branches. The scale bar shows 0.5 estimated substitutions per site and the trees were rooted to the midpoint. (**b**) Whole-genome-based phylogenomic tree generated by VICTOR, using translated ORFs, predicted in the genomic sequences of phages representing 30 groups, clustered using amino acid sequences of MCP. VICTOR’s pseudo-bootstrap values are indicated near to corresponding branches. The scale bar shows 0.05 estimated substitutions per site.

A phylogenetic analysis conducted with amino acid sequences of MCP, Ter and DNAP provided mismatched topologies of trees (Figure 11, Supplementary Figure S7). This can be a result of the complex evolutionary history, accompanied by exchanges of individual genes and genomic modules. The composition of the DNAP tree clades is less similar to that of the MCP and Ter trees; the latter two have more in common, here, suggesting that the lateral exchange of the DNAP gene could occur more often. Some differences in tree topology may be due to the high level of divergence within the large group of φ29-related phages, making it difficult to identify the true evolutionary history.

Genomes of some φ29-related phages and *Lactococcus* phage KSY1 contain two copies of genes encoding the proteins related to φ29 MCP. The corresponding phylogenetic tree indicates close relatedness, and the possible origin of capsid proteins of *Lactococcus* phage KSY1 by gene duplication, but places two predicted capsid proteins of *Clostridium* phage CpV1 (family *Guelinviridae*) in two clades containing other phages possessing only one MCP. This can indicate a duplication of the MCP gene and its subsequent loss during the evolution of φ29-related phages.

The whole-genome-based phylogenomic tree generated by VICTOR (Figure 11b), in some cases, shows patterns of phage distribution similar to trees based on the MCP and Ter sequences. For example, it places the same phages infecting the same Actinobacteria in a distinct clade and indicates relatedness between *Salmonella* phage Astrid and an unclassified phage found in metagenomic sequences, supposedly infecting a Bacteroidia bacterium. The topology of this tree does, however, have its own peculiarities, which can be explained both by the specificity of phage evolution, distinguished by the different evolutionary history of different genomic modules, and by the high level of sequence divergence. According to the results of phylogenetic analysis, cluster c28 is most closely related to cluster c01, which includes phage φ29. A representative of cluster c28, phage Podoviridae sp. ctnWS46, with a similar genome length of 19,654 bp, has very little (1.2%) intergenomic similarity to phage φ29. Comparisons of morphogenesis and replication proteins indicate a clear relationship between these proteins, but the order of a significant proportion of genes differs from φ29, which indicates frequent genetic rearrangements accompanying the evolution of φ29-like phages.

### 2.6. Variations in the Structural Architecture of Morphogenetic Proteins of φ29-Related Phages Infecting Gram-Negative Bacteria

While most of the sequences of known φ29-related viruses found apparently belong to phages infecting Gram-positive bacteria, some of them were experimentally found, or predicted, to infect Gram-negative bacteria. Such isolated phages are related to *Salmonella* phage astrithr of the genus *Astrithrvirus* (e.g., *Acinetobacter* phage Phanie [13], *Pectobacterium* phage DU_PP_III, *Salmonella* phages Astrid, astrithr and assan [114,115]) and were assigned to cluster 23, based on the similarity of MCPs. Phages Astrid, astrithr and assan are very close (intergenomic similarity >95%) [13] and represent one species, *Astrithrvirus astrithr*, classified by the ICTV [12]. Phages DU_PP_III and Phanie have a significant level of intergenomic similarity to phages *Astrithrvirus astrithr* (about 30–40%, [13]), but this value is still less than the genus demarcation threshold. In addition, a search for homologous proteins strongly suggested that singleton cluster 30 contains a putative phage infecting a Bacteroidales bacterium, a metagenome-assembled genome annotated as “Bacteriophage sp. isolate 2692_55609” and further referred to as “2692_55609”, found in human faeces. Genomes of phages assigned to clusters 23 and 30 were significantly smaller than the φ29 genomes, being typically about 11–14 kbp. The structural architecture of morphogenetic proteins of such phages, at least in some cases, can be different from phages that infect Gram-positive bacteria.

**Figure 12:**
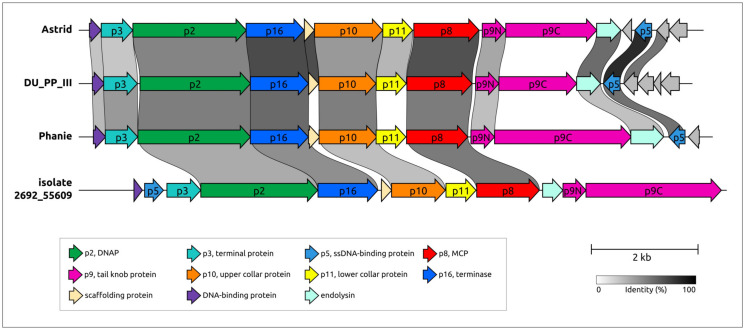
Genetic map of phages belonging to clusters 23 and 30. Phages’ names are as follows: Astrid, *Salmonella* phages Astrid; DU_PP_III, *Pectobacterium* phage DU_PP_III; Phanie, *Acinetobacter* phage Phanie; isolate 2692_55609, Bacteriophage sp. isolate 2692_55609. Annotations based on the results of HMM searches, AF predictions and an earlier-published genomic analysis of phage Phanie [13] are shown in labels. Arrows indicate the direction of transcription. The scale bar indicates the level of identity between genes according to a black–white gradient, the legend for which is shown at the bottom of the figure. The number of clusters and the name of sequences are shown on the left. Genes encoding proteins similar to φ29 (p2, p5, p6, p8–p11, p16) and other proteins are coloured according to the legend.

The genomes of the *Salmonella* phages Astrid, *Pectobacterium* phage DU_PP_III, *Acinetobacter* phage Phanie and phage 2692_55609 have common features that unite them together and distinguish them from the phage φ29 (Figure 12). HMM searches clearly indicate the relationship of the morphogenesis and replication proteins to the proteins of the phage φ29. A list of these proteins includes phage φ29-like DNA polymerase (φ29 p2), terminal protein (counterpart of φ29 p3-like), ssDNA-binding protein (φ29 p5), counterparts of φ29 main virion proteins (MCP, upper and lower collar proteins, tail knob protein, but encoded by two genes, unlike in φ29). The lower collar proteins of these phages are smaller than that of φ29. HHpred and BLAST searches did not reveal genes that encoded tail fibre and tail spike proteins present in some φ29-like phages [9,10]. Interestingly, putative endolysins show resemblance to CHAP domain-containing endolysins and tail-associated lysins of various phages, e.g., an HHpred search revealed clear similarities between the Phanie gp11 and the tail tip lysin of φ29-like phage Andhra, which infects *Staphylococcus epidermidis* [9] (HHpred probability 94%).

**Figure 13:**
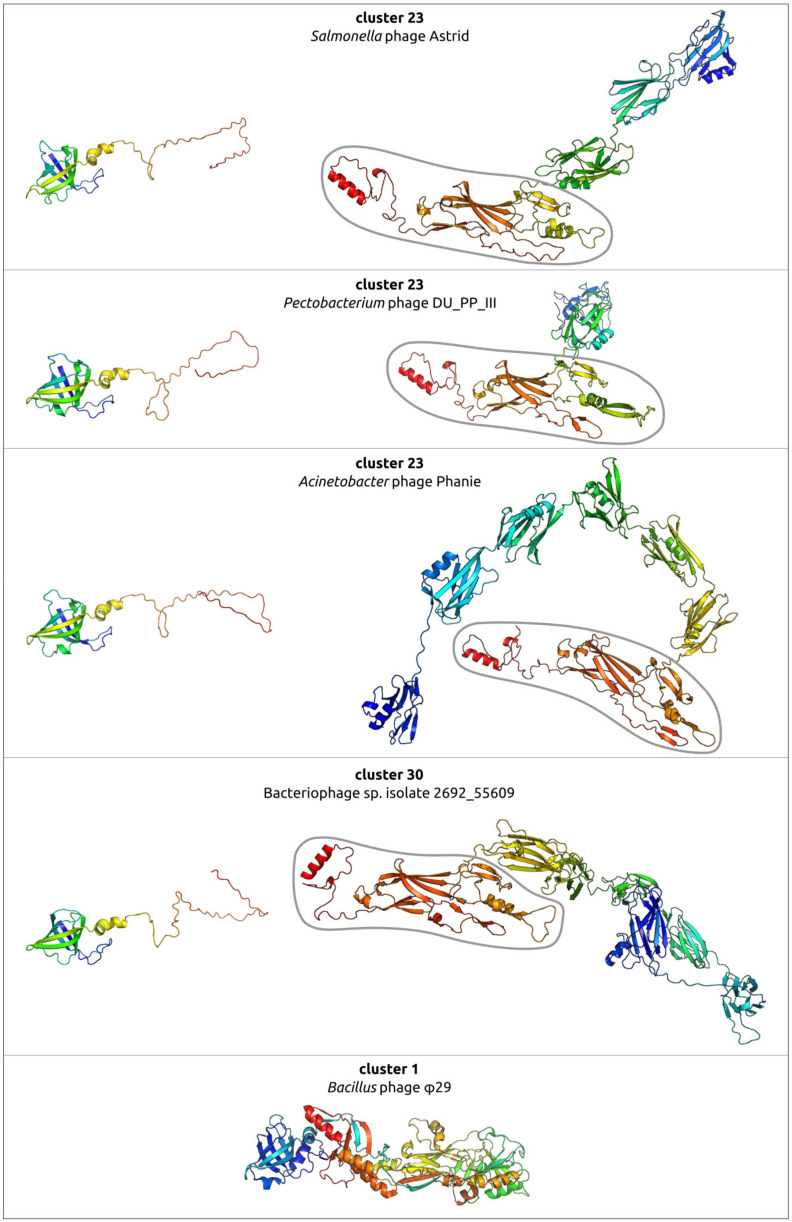
A ribbon diagram of the predicted proteins similar to the N-terminal part of the φ29 tail knob protein and the C-terminal part (major part), coloured using a rainbow gradient scheme, where the N-terminus of the polypeptide chain is coloured blue and the C-terminus is coloured red. The C-terminal fragment of the major part protein, similar to φ29 p9, according to the results of an HHpred search, is contained within a grey lozenge. The image at the bottom of the figure shows the experimentally determined structure of tail knob protein p9 of *Bacillus* phage φ29 (PDB code 5fb5 [49]).

A comprehensive study by Pourcel et al. [13] showed that *Acinetobacter* phage Phanie (genome size 11,885 bp) depends on a helper myophage, and that Phanie virions are non-infectious unless they bind to the contractile tail of the unrelated phage Aci01-1, forming chimeric myoviruses. Perhaps other φ29-related phages infecting Gram-negative bacteria may also follow the strategy of multiplication similar to phage Phanie, since they share some its genomic features, including the structure of tail knob protein. Structure predictions, made in [13], indicated a similarity between the phage Phanie gene product 9 (gp9) and the N-terminal part of tail knob protein p11 of phage φ29 and a similarity between the C-terminal part of Phanie gp10 corresponded to the C-terminal part of φ29 tail knob protein p9; AlphaFold 2 modelling of Phanie gp10 predicted a multidomainal structural architecture (Figure 13) for this protein, where the C-terminal part of Phanie gp10 corresponded to the C-terminal part of φ29 tail knob protein p9. AlphaFold modelling conducted in present study indicates that, counting from the N-terminus, the predicted structure of gp10 contained six domains, followed by the C-terminal part. Neither the BLAST nor the HHpred searches revealed the significant similarity of these domains to any known proteins with an experimentally determined function; the DALI search also found no similar viral structures, but revealed a moderate level of structural similarity (Z-score 5.1–6.1) between all domains of gp10 and an Ig-like non-catalytic domain of L,D-transpeptidase from *Mycobacterium tuberculosis* (PDB code 4Z7A [116]) involved in the biosynthesis of peptidoglycan (PG) [116]. A comparison of predicted structures of putative proteins composing the tail knobs of phages Astrid, DU_PP_III and 2692_55609 indicated the same general plan of their structural architecture, including an obvious similarity of structures of proteins, similar to the N-terminal part of φ29 tail knob proteins, but the predicted proteins, containing the counterpart of the C-terminal part of φ29 tail knob proteins, have a different number of additional Ig-like domains (Figure 13). As was mentioned in the Introduction section, the N-terminal part of φ29 tail knob protein p9 has a fold found in the neck and tail tube proteins of sipho- and myoviruses [19]; the predicted proteins of phages Astrid, DU_PP_III, Phanie and 2692_55609, which are similar to the N-terminal part of φ29 p9, contain the similarly folded domain as well.

**Figure 14:**
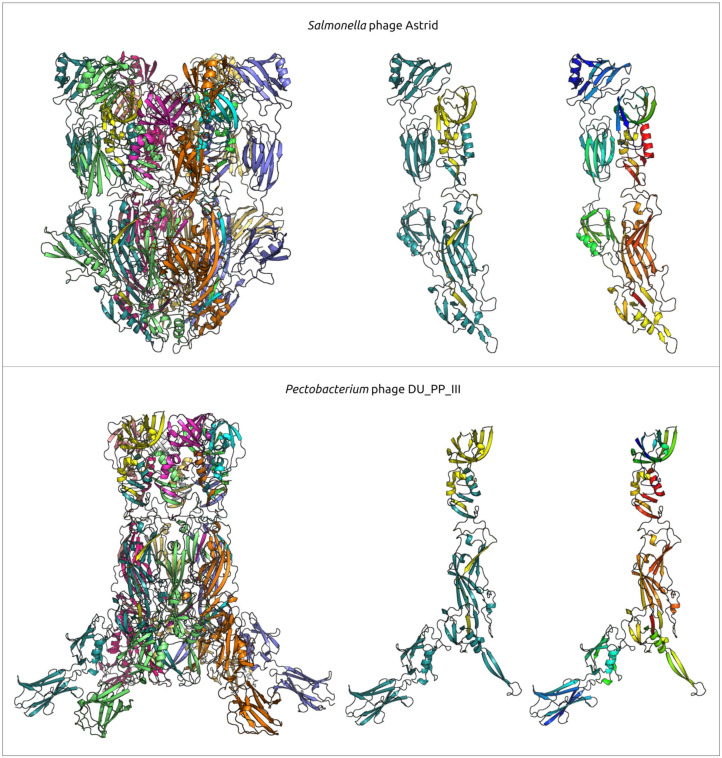
A ribbon diagram of the protein complexes, modelled by AlphaFold3, supposedly corresponding to tail knobs of *Salmonella* phages Astrid and *Pectobacterium* phage DU_PP_III. Left depictions show the full-sized model comprising 12 monomers (each monomer has a different colour). The central depiction shows two closest different monomers, the protein similar to the N-terminal part of the φ29 tail knob protein is coloured yellow, and the protein similar to the C-terminal part is coloured cyan. The right-side depictions show the same monomers as the central one using a rainbow colour gradient, where the N-terminal end of each monomer is blue and the C-terminus is red.

The AlphaFold modelling complexes that uses six copies of each protein, including both supposedly composing the N-part (minor part) of the tail knob of the phages Astrid, DU_PP_II and the C-part (major part), can offer an interesting suggestion for the interactions of the proteins forming the tail knob (Figure 14); both predicted structures contain β-sheets formed by two different monomers. Additional Ig-like domains are located laterally in both structures. The ipTM score of the DU_PP_III model is 0.69, the pTM score is 0.70 and, for the complex containing the Astrid proteins, the scores are lower (0.51 and 0.52, respectively).

### 2.7. Lactococcus Phage KSY1: General Genomic Features and Relatedness to φ29-like Phages

**Figure 15:**
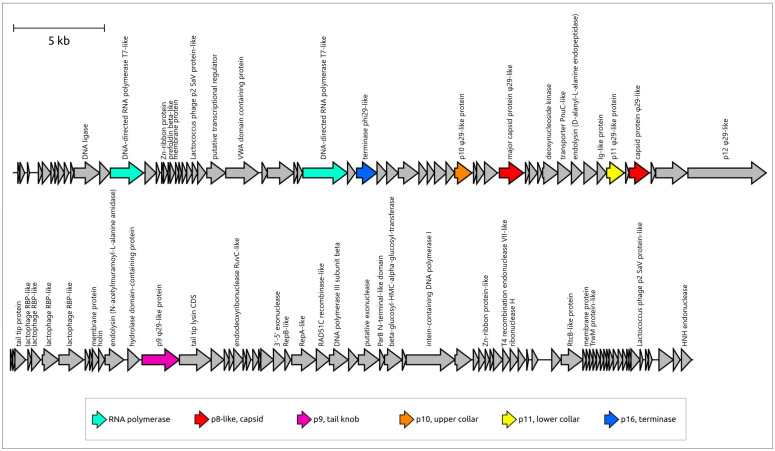
A genetic map of *Lactococcus* phage KSY1. Arrows indicate the direction of transcription. The scale bar indicates the length of the nucleotide sequence. Gene functions are shown in labels and the legend. Genes encoding RNA polymerase and proteins similar to φ29 proteins p8–p11 and p16 are coloured according to the legend.

*Lactococcus* phage KSY1, representing a singleton genus *Chopinvirus*, is a virulent phage infecting *Lactococcus lactis*. Phage KSY1 possesses a unique complex of morphological and genomic features; the phage was reported in 2007 [117], and no closely related phages have been isolated since then. Phage KSY1 is a podovirus of the C3 morphotype and it has a very characteristic appearance, with an elongated capsid (223 nm long, 45 nm wide) and a short tail (32 nm) [117]. HHblits/HHpred searches have clearly indicated the relatedness of the main virion protein to their counterparts in phage φ29. At the same time, the KSY1 genome contains two genes encoding for phage T7-like DNA-dependent RNA polymerase (RNAP) (Figure 15).

According to [117], phage KSY1 has a linear dsDNA genome that is 79,232 bp in length, containing 131 closely packed, predicted open-reading-frames (ORFs) and three tRNA genes. The G + C genome content (35.1%) is close to that of the host (35.3%), indicating the possible long-term coexistence of the phage and its bacterial host. Apparently, the KSY1 genome is blunt-ended and does not contain terminal repeats or redundancy at the genome ends [117].

A BLAST search using the GenBank phage database (GBPHG) indicated that 83 proteins out of 131 have no homologues among phage proteins (E-value < 0.05). The sequences of predicted hydrolases, endolysin gp053a, baseplate proteins, RNA-splicing ligase and proteins with unknown functions have similarities with their counterparts from *Lactococcus* phages, which have a dissimilar main virion, and other proteins. This can indicate that corresponding genes have been acquired via gene exchange. Some other proteins, including endolysin gp073, ribonuclease H and nucleic-acid-processing proteins are more similar to proteins of phages infecting other Gram-positive bacteria.

BLAST and HHpred searches could not find homologues for φ29 DNAP, but found a homologue of DNA polymerase III β subunit (β-clamp) among predicted proteins. It seems that the replication process of KSY1 is quite different to that of phage φ29. At the same time, an HHpred search indicated that ATPAse domain of KSY1 terminase, upper and lower collar proteins and a tail knob-like protein are similar to corresponding proteins of phage φ29 (p16, p10, p11 and p9). Interestingly, a BLAST search could not find similarities in amino acid sequences between the main virion proteins and terminase of φ29-like phages and phage KSY1, except for the tail knob proteins of *Enterococcus*, *Lactoccocus* and other phages belonging to the *Rountreeviridae* family and the tail knob-like protein of phage KSY1. This can indicate a lower rate of evolution of this protein, or it might be the result of gene transfer.

**Figure 16:**
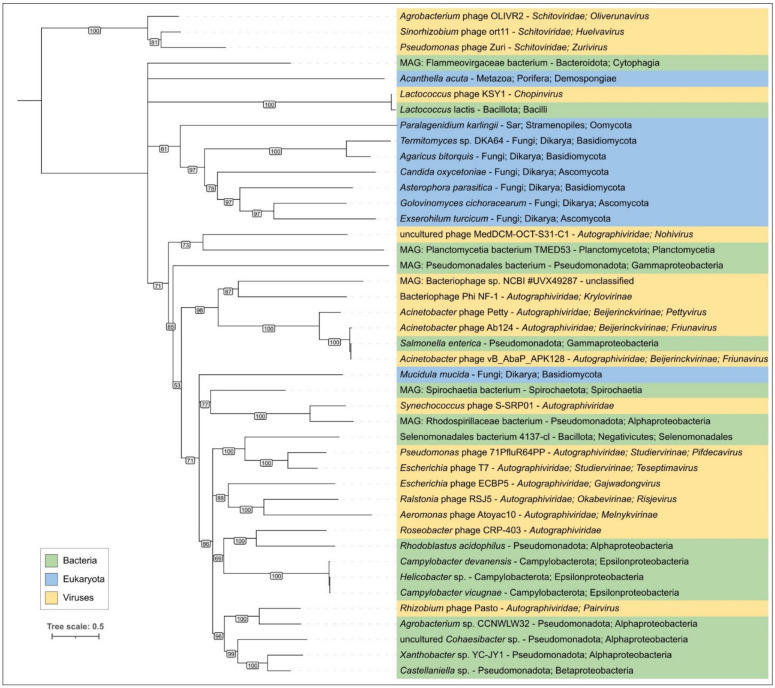
A maximum likelihood phylogenetic tree based on the amino acid sequences of proteins similar to *Lactococcus* phage KSY1 RNA polymerase gp014. NCBI taxonomy is indicated in labels and in the legend. Branches with a bootstrap support lower than 50% have been deleted. Bootstrap values are shown near their branches. The scale bar shows 0.5 estimated substitutions per site and the tree was rooted to the midpoint.

The original paper [117] mentions only one gene of T7-like RNAP predicted in the phage KSY1 genome, but an HHpred search, using the latest databases, indicated that the KSY1 genome contains two genes encoding T7-like RNAP (KSY1 gp014 and gp033). A BLAST search indicated the clear resemblance of KSY1 gp014 to *Autographiviridae* (T7-like) and *Schitoviridae* (N4-like) phages, (the smallest E-value was 3.59 × 10^−7^ for T7-like *Synechococcus* phage S-SRP01), but showed only minor traces of similarity between gp033 and proteins from other phages, (the smallest E-value was 3.17 × 10^−1^ for N4-like phage vB_EamP_Gutmeister infecting *Erwinia*). A BLAST search using the GenBank nr database found homologues of RNAP gp014 in evolutionarily distant groups of cellular organisms and viruses. Phylogenetic analysis placed phage KSY1 gp014 close to a protein from *Lactococcus lactis* and distant from *Autographiviridae* and *Schitoviridae* RNAPs, which, in turn formed distinct clades (Figure 16).

### 2.8. ViPtree Proteome Phylogeny and VIRIDIC Clustering

A phylogenetic tree based on the proteome similarity was obtained using genomic sequences of representatives of each cluster and *Lactococcus* phage KSY1. The tree was obtained by the ViP server that predicts proteins, calculates the level of similarity between proteins, and clusters them using a neighbour-joining algorithm. This approach cannot resolve problems associated with the conflicting evolutionary history of individual proteins obtained by genetic exchanges, but, together with other clustering tools, it is recommended by ICTV [6]. The tree (Supplementary Figure S8) places all φ29-like phages except representatives of cluster 02 (e.g., *Staphylococcus* phage Andhra of the *Rountreeviridae* family), cluster 22 (e.g., unclassified phage plasmids *Enterococcus*) and cluster 27 (*Lactococcus* phage asccphi28) in a distinct clade containing only φ29-like phages. Representatives of phages of clusters 02, 22 and 27 were placed in another big clade, while *Lactococcus* phage asccphi28 (cluster 27) was grouped together with *Lactococcus* phage asccphi28, and representatives of clusters 02 and 22 were placed in a branch containing the φ29-like phages infecting *Enterococcus* and *Staphylococcus*. Importantly, the topology of the tree, constructed by the ViPtree, in the part of deep-rooted clades, was different for different sets of phages, and some relationships were inferred from just a few or one protein, e.g., the ViPtree placed *Lactococcus* phage asccphi28 and *Lactococcus* phage KSY1 in a separate clade, based on the similarity of a fragment of endolysin corresponding to the region 48,427–47,858 bp.

Pairwise intergenomic similarity calculations with VIRIDIC (Supplementary Figure S9) indicated low levels of intergenomic similarity between members of different φ29-like phage clusters. These values were 0–5%, which are well below the genus-delimiting threshold of 70%.

## 3. Discussion

The application of the definition of taxonomic species to the description of evolutionary history of biological objects with chimeric genomes is a difficult task. The limited period of the existence of species and the lability of viral genomes can also make attempts to reconstruct the evolutionary history almost pointless. The processes of genetic exchange seem to be especially pronounced in temperate phages, where the phylogenetic analysis of even the most-conserved proteins (e.g., MCP or terminase) shows a strikingly different topology of the trees [118]. As can be seen from the results of clustering, genomic comparisons and phylogenetic analysis, genetic rearrangements between evolutionarily close groups of phages can also affect φ29-like phages. However, we can try to plot the evolutionary traits of genes encoding separate essential proteins and their stable complexes.

The group of φ29-like phages possesses pronounced unique features in packaging and replication, which are interrelated processes. Phages of this group have a similar architecture of their genomes and similar virion proteins (e.g., capsid and portal proteins). Structural interactions between morphogenesis proteins drives the co-evolution of these proteins and may be one of the reasons for the stability of φ29-like genomes. HMM and BLAST searches using the sequences of the major capsid protein and terminase carried out in this study allowed us to identify relative phages. The results indicate that the set of main virion proteins (p6-p11) is the hallmark feature of φ29-like group, though their sequences may significantly differ from the sequences of other phage proteins. The proteins comprising replication complex and terminase are also characteristic for these phages.

**Figure 17:**
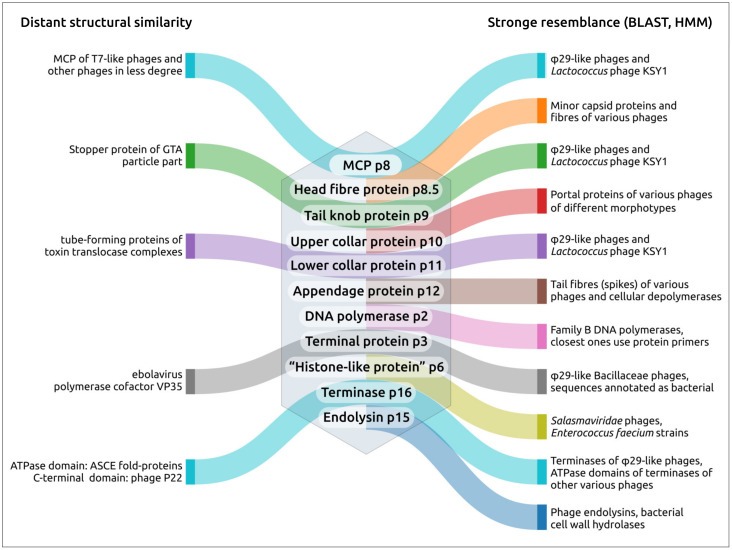
Diagram indicating the close and distant relatedness of φ29 proteins, based on the conducted analyses and earlier-published data.

The present research indicates that the evolution of φ29-like viruses was accompanied by genome rearrangements, gene duplication and loss, and the acquisition of domains of apparently cellular origin. The latter applies even to such a conserved protein as the major capsid protein. Notably, a comparison of the structures of the φ29 major capsid protein (excluding the Ig-like domain presumably acquired by horizontal transfer) showed that the φ29 MCP is more similar to its counterpart from *Autographiviridae* phages than to its counterparts from other groups of *Caudoviricetes* phages (Figure 4 and Figure 17, Supplementary Figure S5). This could be an argument for the evolutionary relatedness of φ29-like and T7-like phages. The unlikeness of amino acid sequences of MCP of φ29-like phages to sequences of MCPs of all other *Duplodnaviria* viruses (except phage KSY1), including T7-like phages, however, which has been confirmed by HMM searches, is a strong indication of the ancient divergence of the φ29-like phage group.

The evolution of φ29-like phages may involve both coevolution with the host and host switching, based on an analysis and comparisons of phylogenetic trees relating to phage and bacterial sequences. Members of φ29-like groups of phages are able to infect a wide range of hosts, and the analysis of metagenomic sequences indicated that, in addition to bacteria of the Bacillales, Clostridia, Enterobacterales and Pseudomonadales orders, these phages might be able to infect representatives of the Bacteroidales order. Perhaps the phage Bacteriophage sp. isolate 2692_55609, representing cluster c30, is the earliest diverged φ29-like phage (among the analysed representatives) which infects Gram-negative bacteria. A phylogenetic analysis of MCP and DNAP indicates that this phage is related to *Amedibacillus* phage AD70P2 (cluster c24). *Amedibacillus* bacteria are phylogenetically distant from Gram-negative bacteria. The origin and evolution of φ29-like phages infecting Gram-negative bacteria is probably linked to host switching followed by modification of some proteins. Particularly, Gram-negative-specific phages have shortened lower collar (tail tube) protein, which may be due to the thinner cell wall of the bacterial host. Also, the recent publication on *Acinetobacter* phage Phanie [13] and the results of this work suggest the distinct composition of the tail knob in phages infecting Gram-negative bacteria. The tail knob of analysed φ29-like phage infecting Gram-negative bacteria appears to be a hexamer, each unit of which consists of two proteins. One of them is located closer to the phage head and corresponds to N-terminal part of φ29 tail knob protein p9, and the second distal protein is analogous to the carboxy-terminal part of φ29 p9. This split of the gene encoding the tail knob protein probably occurred in the ancestor of φ29-like phages infecting Gram-negative bacteria due to genetic exchange, and resulted in the insertion of the fragment encoding Ig-like domains. It has been shown that Ig-like domains can participate in the interaction with carbohydrates on the surface of the bacterial cell [119] and participate in the formation of phage tail appendages and sheaths [120,121]. The results of structural predictions suggest that Ig-like domains are located lateral to the tail tube. Particularly, the AF model of the protein complex of a tail knob of *Pectobacterium* phage DU_PP_III, predicted with a decent quality (ipTM score 0.69, pTM score 0.70), depicts the Ig-like domains down to the phage head as resembling short tail fibres, though with architecture fundamentally different from common trimeric architecture. A less reliable model of the tail knob of *Salmonella* phages Astrid also places the Ig-like domains lateral to the tail tube, but adjacent to the tail tube. *Acinetobacter* phage Phanie contains six such domains in gp11. They resemble the Ig-like non-catalytic domain of L,D-transpeptidase from *Mycobacterium tuberculosis*; hypothetically, these domains are able to exhibit carbohydrate-binding properties. The formation of chimeric virions of phage Phanie and helper myovirus *Acinetobacter* phage Aci01-1 was experimentally demonstrated [13]. Based on TEM images [13], myoviral tail tube is attached distally to the tail of phage Phanie. In a previous work [121], it was suggested that the additional Ig-like domains of a myoviral tail sheath protein can facilitate increasing the stability of the sheath due to protein–protein interactions involving additional domains. It is probable that these domains play an important role in the formation of chimeric virions.

Unexpectedly, HMM searches revealed that a peculiar phage, *Lactococcus* phage KSY1 (*Chopinvirus* KSY1) [117], which is very much not like φ29 in genome size and content, has φ29-like terminase and the virion proteins, including homologues of φ29 MCP and non-RBP tail proteins. Perhaps φ29-like phages are just a part of an even larger clade of evolutionarily related bacteriophages. Seemingly, *Lactococcus* phage KSY1 acquired genes of adsorption apparatus from other phages infecting *Lactococcus* or related bacteria, but the source of the genes involved in transcription and replication requires additional research. However, a sequence search has clearly indicated the relatedness of *Chopinvirus*, *Autographiviridae* and *Schitoviridae* RNA polymerases, and phylogenetic analysis has indicated that the RNAPs of all three taxa belong to different clades. It will be interesting to see if the *Lactococcus* phage KSY1 can help find the link between the T7- and φ29-like viruses. In spite of having transcription apparatus resembling T7-like phages [117], the genome of phage KSY1 contains neither T7-like DNA polymerase nor φ29-like DNAP. According to the results of phylogenetic analysis, the latter is related to the DNAP of tectiviruses.

The initial steps of phage infection include the attachment and injection of phage DNA into the bacterial host [122]. Bacterial cells typically contain peptidoglycan, which acts as an obstacle to phage infection; phage φ29 must penetrate an approximately 250 Å thick outer peptidoglycan cell wall [48]. Different phages overcome this obstacle in different ways. Tectiviruses (class *Tectiliviricetes*) use a tubular structure made of a viral membrane that is formed after adsorption to the host–cell to deliver the phage genome into the host–cell [123]. Tailed phages (class *Caudoviricetes*) can use their tails, which are made of proteins, as a genome delivery device. *Caudoviricetes* tails can be one of three types, namely short noncontractile (podoviruses), long noncontractile (siphoviruses) and long contractile (myoviruses). A structural analysis of tail proteins reveals the evolutionary relatedness of the long tails of tailed phages, myoviruses and siphoviruses, as well as their common evolutionary origin with secretion systems of type VI (T6SS) and bacteriocins [124,125,126]. It is difficult to say which functions were performed by the last common ancestor of these nanomachines, and the details of their origin need clarification, but some observations indicate that T6SSs might represent an early diverged branch, whereas bacteriocins could appear multiple times in different organisms and have proteins similar to the tail proteins of phages that infect these organisms [121].

Although some evolutionary relatives of long-tailed phages are known, the details of the evolution of short-tailed phages are less understood. Actually, some domains of phage structural proteins, especially those participating in adsorption and cell-wall degradation, can have a resemblance with proteins or protein domains of cellular origin, as well as with proteins of viral origin, even those encoded in genomes of phages belonging to distant viral groups, but these cases are apparently the result of horizontal transfer [99]. From this point of view, the results of the current study, which indicate the resemblance of the φ29 tail tube formed by lower collar protein p11 and tail tubes of toxin translocase complexes of Gram-positive bacteria, could offer a partial explanation of the origin of the tail of φ29-like phages. A search for similar structures using non-RBP tail proteins of φ29, excluding upper collar (portal) protein, which is common for all tailed phages and herpesviruses, and which could be considered to be part of the tail head (capsid), did not find similar proteins among podoviruses belonging to well-studied groups of T7-like phages (family *Autographiviridae*) and N4-like phages (family *Schitoviridae*). A previous publication [19] indicates that the amino-terminal domain of the tail knob proteins of a φ29-like phage C1 infecting *Streptococcus*, and apparently of φ29 itself, has a fold found in tube-forming proteins of sipho- and myoviruses phages, indicating evolutionary ties between long-tailed and short-tailed phages. A similar fold was found in phage and herpesvirus proteases [127], as well as in the tail stopper protein of the GTA of *Rhodobacter capsulatus*, structurally resembling p11 according to DALI (Section 2.2.3). However, this fold seems to be missing the protein structures of another podoviral tail tube, namely of phage T7 (see, for example, PDB #6R21 [128]). Perhaps the morphological classification of podoviruses needs some clarification, since their tails may have different origin and architecture.

Koonin et al. [80] suggested the importance of capturing host genes by viruses during their evolution. This suggestion is consistent with the assumption that the ancestor of tailed phages adapted different bacterial transport systems to deliver its genome into the host–cell. Future myoviruses and siphoviruses could adapt an ancestor of T6SS, and the ancestor(s) of modern φ29-like phages could utilise parts of bacterial toxin translocase complexes. It is also possible that, during evolution, phages might change their morphology. The results of recent studies by Pourcel et al. [13], for example, which show the formation of a chimeric virion, may indicate the possibility of the emergence of new chimeric viruses. The first step of such a transformation could be changes in protein structures, which lead to the appearance of stable chimeric virions, with subsequent steps involving genetic exchanges that enable the formation of a chimeric genome. Similarly, one could imagine that the ancestor of the T6SS or pore-forming translocase complex and a tailless phage gave rise to tailed phages. Moreover, hypothetically, viral ancestors of tailed phages might perform a successful penetration and infection function using mainly cellular machinery.

In addition, the cellular origin of phage proteins may help to shed light on the origin of the packaging complex of φ29-like phages that contain unusual packaging RNA. The origin of the C-terminal domain of phage φ29 terminase (CTD) resembling an RNase H nuclease fold may be linked to the loss of the nuclease function of a large subunit of phage terminase that resulted in the formation of the simplified vestigial nuclease fold CTD [59]. The authors also suggest the further simplification of CTD, resulting in an experimentally shown loss of ability to bind the connector and the replacement of this function by pRNA [59]. Alternatively, the authors propose the origin of pRNA based on the RNA-world hypothesis, where RNA was used as both genetic material and functional macromolecules. In this case, pRNA is a relict that may be left conserved in φ29-like phages as descendants of ancient phages, while other phages have transferred this function to proteins [59]. We would also propose a third hypothesis, in which pRNA is a vestige left over from a cellular complex adapted by phages for their purposes and subsequently lost. The similarity between the ATPase domain of φ29 terminase and the DEAD/DEAH-box RNA helicases and other proteins characterised by the ASCE fold may indicate a cellular origin for terminase. HMM and DALI searches have indicated a clear similarity between the φ29 terminase and RNA-dependent nucleoside triphosphatases, which can employ RNA-stimulated nucleoside triphosphatase activity to remodel RNA or ribonucleoprotein complexes [129]. Cellular proteins with ATPase and nuclease domains are also known, e.g., DNA2 nuclease/helicase [130].

Summarising the aforesaid, the group of φ29-like phage clearly differs from other phage groups. It has much in common, though it has variations in genomes, bacterial hosts and infection mechanisms. A search for φ29-like and related viruses conducted for the present study could not find many members of this group, since it employed the HMM-based method on a limited database. The search did, however, identify more than 2000 sequences, indicating the great abundance of these viruses. The prevalence of φ29-related viruses, the complex of unique features, including the characteristic morphology and replication processes, and their distinctive origin, including the early divergence of conserved phage proteins, suggest the possibility of delineating this group as a separate taxon. Perhaps it should be ranked at the level of an order or higher.

## 4. Materials and Methods

### 4.1. Data Collection and Database Construction

Phage genomic sequences were downloaded from NCBI GenBank PHG and NCBI Genome databases (https://www.ncbi.nlm.nih.gov, accessed on 10 January 2024). Downloaded sequences were checked for the presence of duplicates and small fragments that did not contain viral structural, replication or lysis genes, using the Geneious Prime version 2023.2.1 (Biomatters, Inc., Auckland, New Zealand) tools and a BLAST search [131] of the NCBI nt database. Protein structures were downloaded from the Research Collaboratory for Structural Bioinformatics Protein Data Bank (RCSB PDB) (https://www.rcsb.org, accessed on 27 October 2023). Virion structure was downloaded from the Electron Microscopy Data Bank (EMDB) (https://www.ebi.ac.uk/emdb/, accessed on 1 March 2024). BLAST databases were constructed using the “makeblastdb” BLAST command.

### 4.2. Protein Structure Modelling

Protein structures were predicted using AlphaFold (AF); protein complexes were modelled using AlphaFold version 3 [132] (https://alphafoldserver.com/, accessed on 1 September 2024), and other protein structures were predicted with AlphaFold version 2.3.2 [133] using full databases and the command line parameters “--monomer” (for monomeric protein) or “--multimer” (for protein complexes). The best-ranked structures were selected for further study. Protein structures were aligned and visualised using Pymol version 2.5.4 (Schrödinger Inc., New York, NY, USA).

### 4.3. Functional Annotation

Phage genomes were partially reannotated with the assistance of Glimmer 3.0.2 [134], which was used for open-reading-frame (ORF) detection. The functions of the major capsid protein were predicted using a BLAST homology search and an HHpred search using PDB70_mmcif_2023-06-18, PfamA-v35, UniProt-SwissProt-viral70_3_Nov_2021 and NCBI_Conserved_Domains(CD)_v3.19 databases [135]. The functions of tail proteins’ domains were predicted using an HHpred search and a DALI search [136].

### 4.4. Phylogenetic Analysis, Structural Comparison and VIRIDIC Analysis

Multiple alignments of primary amino acid sequences were obtained with MAFFT version 7.48 [40], with default settings, and using the L-INS-i algorithm. A phylogenetic analysis based on the sequence alignments of viral sequences was performed using IQ-TREE version 2.2.5 [137] and the “--alrt 10000 -B 5000” command line parameters. These parameters assume the use of ModelFinder [138] for finding the best substitution model. The resulting consensus trees with bootstrap support values (10,000 replicas) were visualised using iTOL version 6 [139]. Protein structural similarity was assessed using the DALI Z-score [140]. A heatmap based on the pairwise structural similarity of viral capsid proteins and encapsulin major shell proteins, measured by DALI Z-score, was generated using DALI DaliLite.v5. Intergenomic comparison was performed using VIRIDIC version 1.1 [141], applying the default settings. A VICTOR whole-genome phylogenetic tree was generated by running VICTOR on amino acid data, using the D6 formula [142]. A phylogenetic analysis based on the concatenated alignments of essential bacterial genes was performed using UBCG2 [143]. Bacterial genomic sequences were downloaded from NCBI Genome databases (https://www.ncbi.nlm.nih.gov, accessed on 10 January 2024). Genome comparison and genetic maps were made with clinker [144].

### 4.5. Similarity Clustering Analysis

Based on checked and revised annotations of phage genomes, two separate databases were constructed, using major capsid protein (MCPs) and terminase (Ter) predicted sequences. DIAMOND version 2.1.9 [145] was employed as a sequence aligner for proteins, to obtain sequence identity percentages, after which Gephi version 0.10.1 [146] was applied to construct networks based on the identity measure. The networks were visualised using the Force Atlas 2 layout algorithm (tolerance = 0.6, approximate repulsion = true, approximation = 0.8, scaling = 4.0, stronger gravity = true, gravity = 0.04, prevent overlap = true, normalised edge weights = true) [147] and clustered using the community detection algorithm—Modularity [148] (resolution = 2.0 [149]).

## Figures and Tables

**Table 1:** General genome features of sequences representing 30 groups of φ29-related viruses obtained by MCP clustering, and *Lactococcus* phage KSY1.

Cluster	Name	Sequence Length, bp	% GC	Host	NCBI Taxonomy	Isolation Source, NCBI
01	*Bacillus* phage phi29	19,282	40.0%	*Bacillus subtilis*	*Viruses; Duplodnaviria; Heunggongvirae; Uroviricota; Caudoviricetes; Salasmaviridae; Picovirinae; Salasvirus; Salasvirus phi29*	
02	*Staphylococcus* phage Andhra	18,546	29.8%	*Staphylococcus epidermidis*	*Viruses; Duplodnaviria; Heunggongvirae; Uroviricota; Caudoviricetes; Rountreeviridae; Rakietenvirinae; Andhravirus; Andhravirus andhra*	
03	*Arthrobacter* phage Anjali	19,679	59.4%	*Arthrobacter globiformis*	*Viruses; Duplodnaviria; Heunggongvirae; Uroviricota; Caudoviricetes; Anjalivirus; Anjalivirus anjali*	Soil
04	*Caudoviricetes* sp. isolate ctLx22	14,372	35.7%	Bacillota (suggested)	*Viruses; Duplodnaviria; Heunggongvirae; Uroviricota; Caudoviricetes*	Human Metagenome
05	*Caudoviricetes* sp. isolate ctOKO3	17,065	35.7%	*Clostridium* (suggested)	*Viruses; Duplodnaviria; Heunggongvirae; Uroviricota; Caudoviricetes*	Human Metagenome
05	*Methanobrevibacter* sp. isolate RGIG9610	16,264	34.6%	Bacillota (suggested)	Archaea; Euryarchaeota; Methanomada group; Methanobacteria; Methanobacteriales; Methanobacteriaceae; *Methanobrevibacter*	Ruminant gastrointestinal tract
06	*Caudoviricetes* sp. isolate ct1aZ12	15,307	42.6%	Bacillota (suggested)	*Viruses; Duplodnaviria; Heunggongvirae; Uroviricota; Caudoviricetes*	Human Metagenome
06	*Clostridium* phage CS229P1	16,473	37.8%	*Clostridium symbiosum*	*Viruses; Duplodnaviria; Heunggongvirae; Uroviricota; Caudoviricetes*	
07	*Actinomyces* phage Av-1	17,171	49.5%	*Actinomyces naeslundii*	*Viruses; Duplodnaviria; Heunggongvirae; Uroviricota; Caudoviricetes; Dybvigvirus; Dybvigvirus Av1*	
08	Human gut phage 3064_54045	15,891	51.2%	*Bifidobacterium* (suggested)	*Viruses*	Faeces
09	*Caudoviricetes* sp. isolate ctciW69	14,784	41.5%	*Clostridium* (suggested)	*Viruses; Duplodnaviria; Heunggongvirae; Uroviricota; Caudoviricetes*	Human Metagenome
10	*Bifidobacterium* phage BD811P1	17,657	54.8%	*Bifidobacterium dentium*	*Viruses; Duplodnaviria; Heunggongvirae; Uroviricota; Caudoviricetes*	Municipal sewage
11	Bacteriophage sp. isolate ct4VF5	16,226	38.7%	Clostridia (suggested)	*Viruses*	Human Metagenome
12	*Caudoviricetes* sp. isolate ctFJX15	12,830	38.7%	Actinomycetia (suggested)	*Viruses; Duplodnaviria; Heunggongvirae; Uroviricota; Caudoviricetes*	Human Metagenome
13	Virus sp. ctxAI8	13,382	49.8%	Clostridia (suggested)	*Viruses*	Human Metagenome
14	*Caudoviricetes* sp. isolate ctPQG16	12,323	53.5%	Gram-positive (suggested)	*Viruses; Duplodnaviria; Heunggongvirae; Uroviricota; Caudoviricetes*	Human Metagenome
15	Bacteriophage sp. isolate ctLK83	14,962	40.1%	Bacillota (suggested)	*Viruses*	Human Metagenome
16	*Caudoviricetes* sp. isolate ctF653	15,970	41.3%	Clostridia (suggested)	*Viruses; Duplodnaviria; Heunggongvirae; Uroviricota; Caudoviricetes*	Human Metagenome
17	*Caudoviricetes* sp. isolate ctjx911	13,168	37.8%	Bacilli (suggested)	*Viruses; Duplodnaviria; Heunggongvirae; Uroviricota; Caudoviricetes*	Human Metagenome
18	*Bifidobacterium* phage BadAztec2	18,689	48.7%	*Bifidobacterium asteroides*	*Viruses; Duplodnaviria; Heunggongvirae; Uroviricota; Caudoviricetes; Badaztecvirus; Badaztecvirus badaztec1*	Honeybee gut
19	*Streptococcus* phage Cp-1	19,343	38.8%	*Streptococcus pneumoniae*	*Viruses; Duplodnaviria; Heunggongvirae; Uroviricota; Caudoviricetes; Madridviridae; Cepunavirus; Cepunavirus Cp1*	
20	*Caudoviricetes* sp. isolate ctsL82	13,451	32.4%	Clostridia (suggested)	*Viruses; Duplodnaviria; Heunggongvirae; Uroviricota; Caudoviricetes*	Human Metagenome
21	*Clostridium beijerinckii* DSM 6423 plasmid III	16,762	28.4%	*Clostridium beijerinckii*	Bacteria; Bacillota; Clostridia; Eubacteriales; Clostridiaceae; *Clostridium*	
21	*Clostridium* phage HM2	17,470	29.4%	*Clostridium saccharoperbutylacetonicum*	*Viruses; Duplodnaviria; Heunggongvirae; Uroviricota; Caudoviricetes*	
21	*Methanobrevibacter* sp. isolate RGIG9632	16,579	34.2%	Clostridia (suggested)	Archaea; Euryarchaeota; Methanomada group; Methanobacteria; Methanobacteriales; Methanobacteriaceae; *Methanobrevibacter*	Gut metagenome
22	*Enterococcus faecalis* BE32 plasmid pBE32_2	29,025	32.5%	*Enterococcus faecalis*	Bacteria; Bacillota; Bacilli; Lactobacillales; Enterococcaceae; *Enterococcus*	
22	*Enterococcus* phage EF62phi	30,505	32.7%	*Enterococcus faecalis*	*Viruses; Duplodnaviria; Heunggongvirae; Uroviricota; Caudoviricetes*	
23	*Salmonella* phage Astrid	11,713	39.8%	*Salmonella enterica*	*Viruses; Duplodnaviria; Heunggongvirae; Uroviricota; Caudoviricetes; Astrithrvirus; Astrithrvirus astrithr*	Wastewater
24	*Amedibacillus* phage AD70P2	12,320	26.7%	*Amedibacillus* sp.	*Viruses; Duplodnaviria; Heunggongvirae; Uroviricota; Caudoviricetes; Guelinviridae*	Municipal sewage
25	*Caudoviricetes* sp. isolate ct2Eo1	13,822	45.1%	Bacillota (suggested)	*Viruses; Duplodnaviria; Heunggongvirae; Uroviricota; Caudoviricetes*	Human Metagenome
26	Caudovirales phage NCBI #CAOBPP	13,791	37.5%	Bacillota (suggested)	*Viruses; Duplodnaviria; Heunggongvirae; Uroviricota; Caudoviricetes; environmental samples*	Human faeces
27	*Lactococcus* phage asccphi28	18,762	33.7%	*Lactococcus lactis*	*Viruses; Duplodnaviria; Heunggongvirae; Uroviricota; Caudoviricetes; Salasmaviridae*	
28	Podoviridae sp. ctnWS46	19,654	32.3%	*Streptococcus* (suggested)	*Viruses; Duplodnaviria; Heunggongvirae; Uroviricota; Caudoviricetes*	Human Metagenome
29	*Clostridium* phage CpV1	16,748	30.5%	*Clostridium perfringens*	*Viruses; Duplodnaviria; Heunggongvirae; Uroviricota; Caudoviricetes; Guelinviridae; Denniswatsonvirinae; Capvunavirus; Capvunavirus CpV1*	
30	Bacteriophage sp. isolate 2692_55609	12,148	42.5%	Bacteroidales (suggested)	*Viruses*	Faeces

## Data Availability

The original contributions presented in the study are included in the article/Supplementary Materials; further inquiries can be directed to the corresponding authors.

## Supplementary Materials

The following supporting information can be downloaded at: https://www.mdpi.com/article/10.3390/ijms251910838/s1. Ref. [150] is cited in the Supplementary Materials.
