# Supplementary material for: Origin, Evolution and Diversity of φ29-like Phages—Review and Bioinformatic Analysis
Source: Int J Mol Sci. 2024 Oct 9;25(19):10838. doi: 10.3390/ijms251910838 (PMC11476376; doi:10.3390/ijms251910838)
Supplement: Supplementary file 1 [file ijms-25-10838-s001.zip › ijms-3229428 Figures S1-S9.pdf]

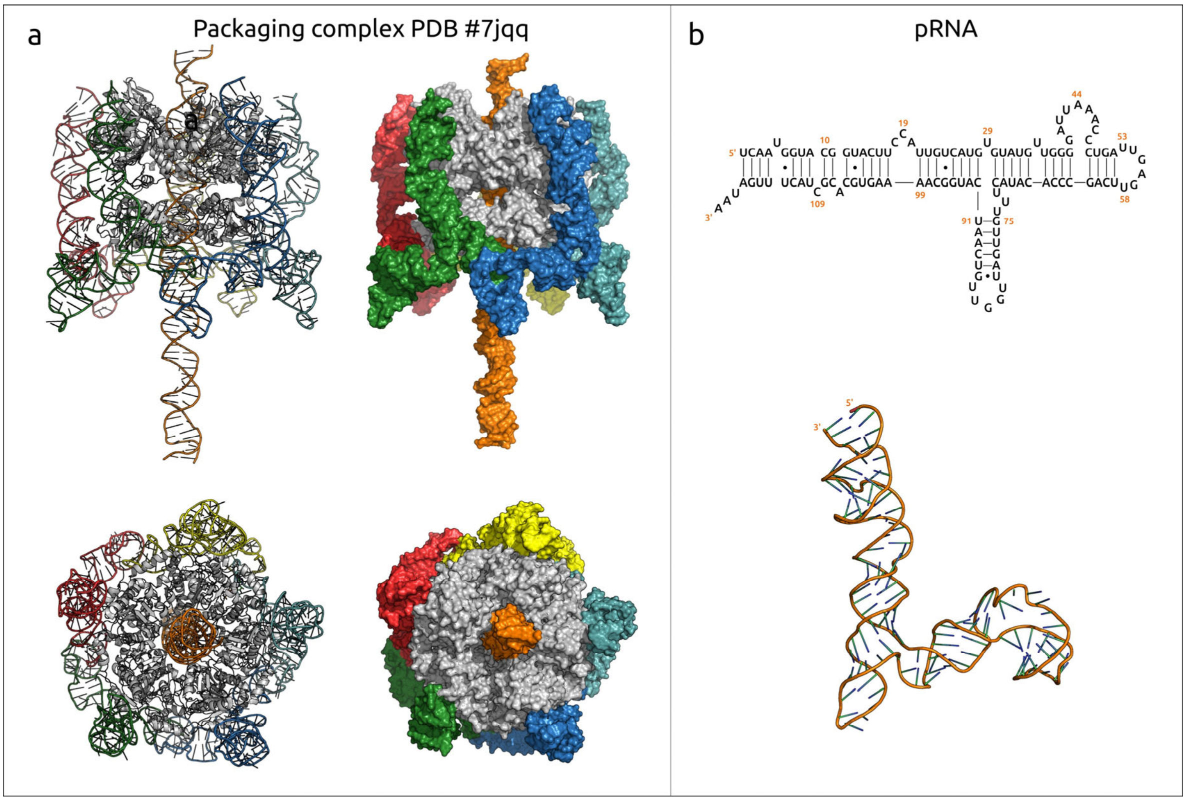

Figure S1. (a) Terminase (all monomers are coloured grey) and pRNA (each monomer has a different colour) in the φ29 packaging motor; ribbon and surface-rendered representations, including top view. Phage DNA is coloured orange. The image was made using PDB structure 7jqq [52]. (b) pRNA secondary structure, according to Ding et al. (2011) [150], and tertiary structure, based on PDB structure 7jqq [52].

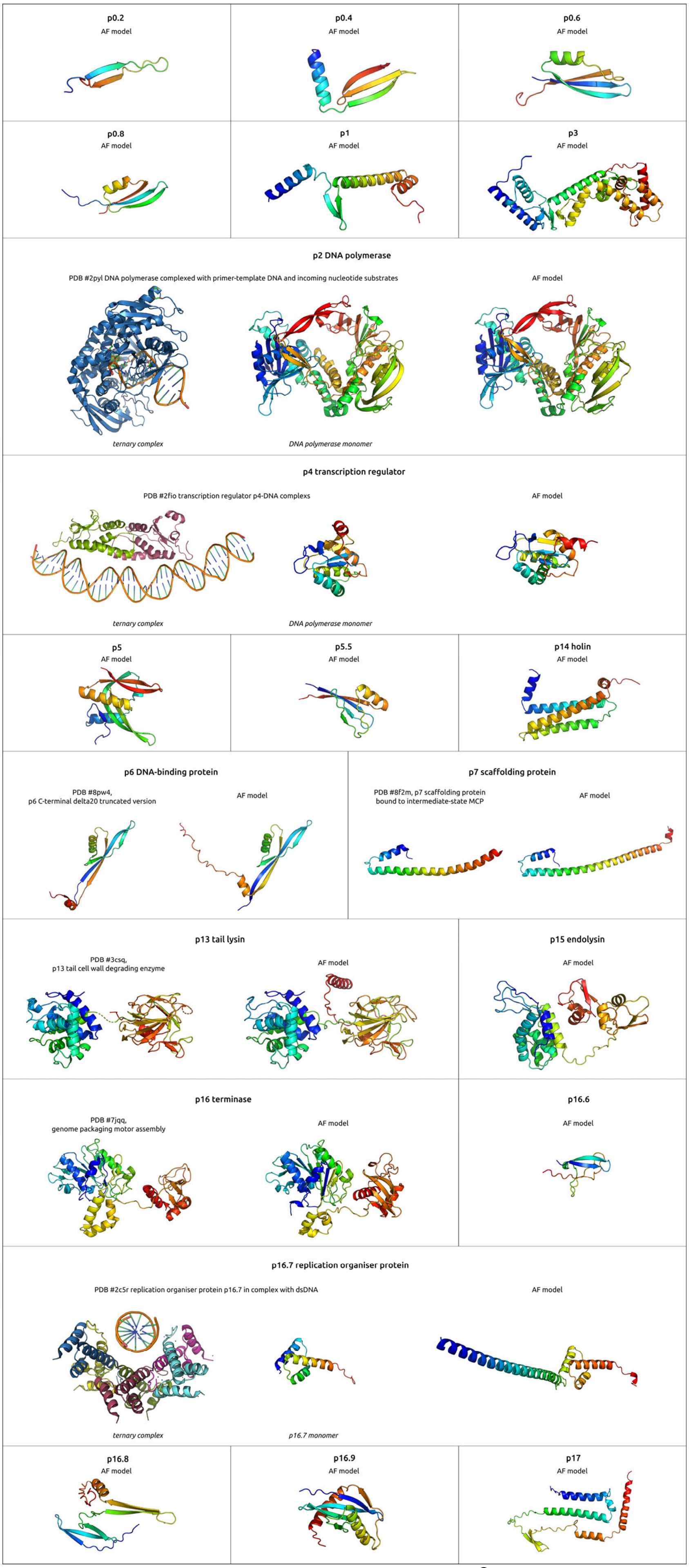
p0.2
AF model
p0.4
AF model
p0.6
AF model
p0.8
AF model
p1
AF model
p3
AF model
p2 DNA polymerase
PDB #2pyl DNA polymerase complexed with primer-template DNA and incoming nucleotide substrates
AF model
ternary complex
DNA polymerase monomer
p4 transcription regulator
PDB #2fio transcription regulator p4-DNA complexs
AF model
ternary complex
DNA polymerase monomer
p5
AF model
p5.5
AF model
p14 holin
AF model
p6 DNA-binding protein
PDB #8pw4,
p6 C-terminal delta20 truncated version
AF model
p7 scaffolding protein
PDB #8f2m, p7 scaffolding protein
bound to intermediate-state MCP
AF model
p13 tail lysin
PDB #3csq,
p13 tail cell wall degrading enzyme
AF model
p15 endolysin
AF model
p16 terminase
PDB #7jqq,
genome packaging motor assembly
AF model
p16.6
AF model
p16.7 replication organiser protein
PDB #2c5r replication organiser protein p16.7 in complex with dsDNA
AF model
ternary complex
p16.7 monomer
p16.8
AF model
p16.9
AF model
p17
AF model

Figure S2. Experimentally derived structures and AF models of φ29 proteins. In ternary complexes, different components are distinguished with different colours. Separate monomers are shown using a rainbow colour gradient, where the N-terminal end is blue and the C-terminus is red.

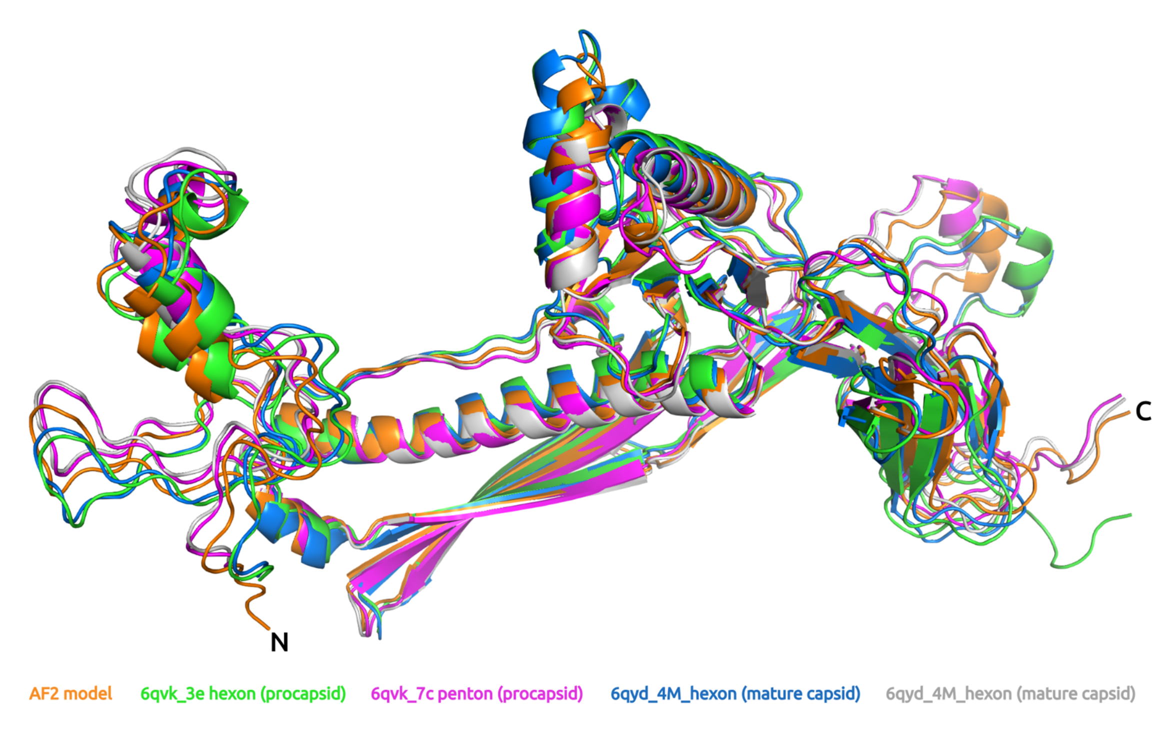

Structure 1	Structure 2	Superimposition
6QVK_7c penton	6QVK_3e hexon	RMSD 3.122 Å
6QVK_9a penton	6QVK_3e hexon	RMSD 3.201 Å
6QYD_4M hexon	6QVK_3e hexon	RMSD 0.779 Å
6QYD_5I hexon	6QVK_3e hexon	RMSD 1.604 Å
6QYD_7O penton	6QVK_3e hexon	RMSD 3.260 Å
6QYD_9a penton	6QVK_3e hexon	RMSD 3.205 Å
AF2 model	6QVK_3e hexon	RMSD 2.306 Å
AF2 model	6QVK_7c penton	RMSD 1.213 Å
AF2 model	6QYD_4M hexon	RMSD 1.898 Å
AF2 model	6QYD_5I hexon	RMSD 0.931 Å
AF2 model	6QYD_7O penton	RMSD 1.100 Å
AF2 model	6QYD_9a penton	RMSD 1.214 Å

Figure S3. Superposition as predicted by AF and experimentally derived structures of major capsid protein. PDB codes, including chain numbers, are indicated in labels.

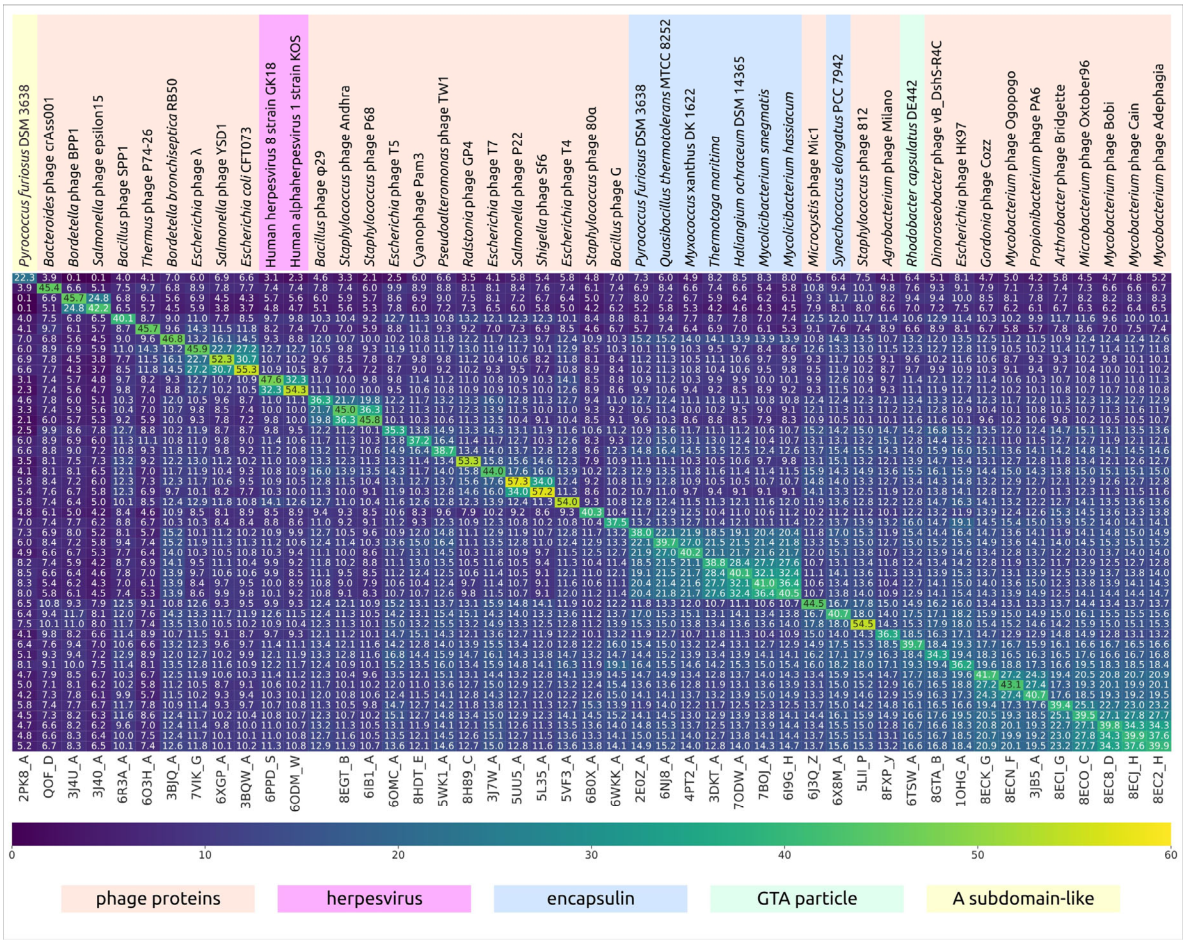

Figure S4. Heatmap with numerical values based on the pairwise structural similarity of viral capsid proteins and encapsulin major shell proteins, as measured by DALI Z-scores.

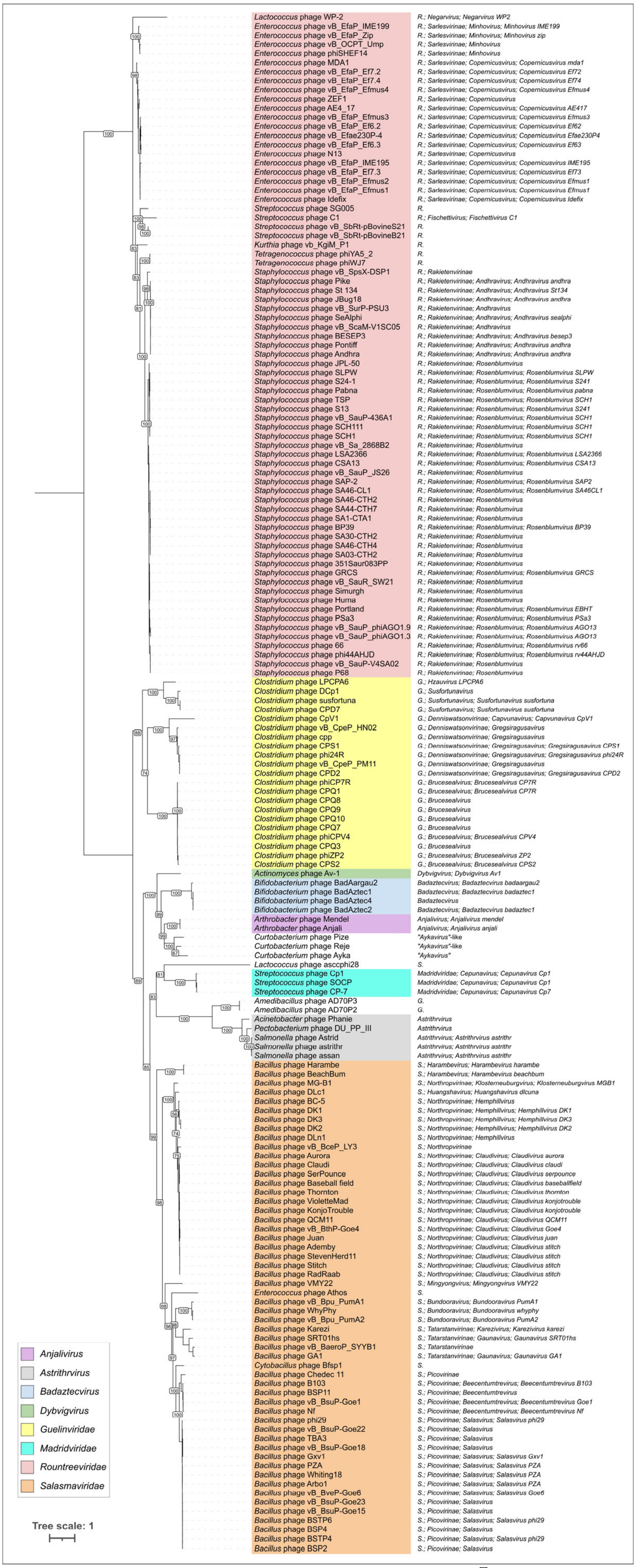

Lactococcus phage WP-2 R.; Negarvirus; Negarvirus WP2
Enterococcus phage vB_EfaP_IME199 R.; Sarlesvirinae; Minhovirus; Minhovirus IME199
Enterococcus phage vB_EfaP_Zip R.; Sarlesvirinae; Minhovirus; Minhovirus zip
Enterococcus phage vB_OCPT_Ump R.; Sarlesvirinae; Minhovirus
Enterococcus phage phiSHEF14 R.; Sarlesvirinae; Minhovirus
Enterococcus phage MDA1 R.; Sarlesvirinae; Copernicusvirus; Copernicusvirus mda1
Enterococcus phage vB_EfaP_Ef7.2 R.; Sarlesvirinae; Copernicusvirus; Copernicusvirus Ef72
Enterococcus phage vB_EfaP_Ef7.4 R.; Sarlesvirinae; Copernicusvirus; Copernicusvirus Ef74
Enterococcus phage vB_EfaP_Efmus4 R.; Sarlesvirinae; Copernicusvirus; Copernicusvirus Efmus4
Enterococcus phage ZEF1 R.; Sarlesvirinae; Copernicusvirus
Enterococcus phage AE4_17 R.; Sarlesvirinae; Copernicusvirus; Copernicusvirus AE417
Enterococcus phage vB_EfaP_Efmus3 R.; Sarlesvirinae; Copernicusvirus; Copernicusvirus Efmus3
Enterococcus phage vB_EfaP_Ef6.2 R.; Sarlesvirinae; Copernicusvirus; Copernicusvirus Ef62
Enterococcus phage vB_Efae230P-4 R.; Sarlesvirinae; Copernicusvirus; Copernicusvirus Efae230P4
Enterococcus phage vB_EfaP_Ef6.3 R.; Sarlesvirinae; Copernicusvirus; Copernicusvirus Ef63
Enterococcus phage N13 R.; Sarlesvirinae; Copernicusvirus
Enterococcus phage vB_EfaP_IME195 R.; Sarlesvirinae; Copernicusvirus; Copernicusvirus IME195
Enterococcus phage vB_EfaP_Ef7.3 R.; Sarlesvirinae; Copernicusvirus; Copernicusvirus Ef73
Enterococcus phage vB_EfaP_Efmus2 R.; Sarlesvirinae; Copernicusvirus; Copernicusvirus Efmus1
Enterococcus phage vB_EfaP_Efmus1 R.; Sarlesvirinae; Copernicusvirus; Copernicusvirus Efmus1
Enterococcus phage Idefix R.; Sarlesvirinae; Copernicusvirus; Copernicusvirus Idefix
Streptococcus phage SG005 R.
Streptococcus phage C1 R.; Fischettivirus; Fischettivirus C1
Streptococcus phage vB_SbRt-pBovineS21 R.
Streptococcus phage vB_SbRt-pBovineB21 R.
Kurthia phage vb_KgiM_P1 R.
Tetragenococcus phage phiYA5_2 R.
Tetragenococcus phage phiWJ7 R.
Staphylococcus phage vB_SpsX-DSP1 R.; Rakietenvirinae
Staphylococcus phage Pike R.; Rakietenvirinae; Andhravirus; Andhravirus andhra
Staphylococcus phage St 134 R.; Rakietenvirinae; Andhravirus; Andhravirus St134
Staphylococcus phage JBug18 R.; Rakietenvirinae; Andhravirus; Andhravirus andhra
Staphylococcus phage vB_SurP-PSU3 R.; Rakietenvirinae; Andhravirus
Staphylococcus phage SeAlphi R.; Rakietenvirinae; Andhravirus; Andhravirus sealphi
Staphylococcus phage vB_ScaM-V1SC05 R.; Rakietenvirinae; Andhravirus
Staphylococcus phage BESEP3 R.; Rakietenvirinae; Andhravirus; Andhravirus besep3
Staphylococcus phage Pontiff R.; Rakietenvirinae; Andhravirus; Andhravirus andhra
Staphylococcus phage Andhra R.; Rakietenvirinae; Andhravirus; Andhravirus andhra
Staphylococcus phage JPL-50 R.; Rakietenvirinae; Rosenblumvirus
Staphylococcus phage SLPW R.; Rakietenvirinae; Rosenblumvirus; Rosenblumvirus SLPW
Staphylococcus phage S24-1 R.; Rakietenvirinae; Rosenblumvirus; Rosenblumvirus S241
Staphylococcus phage Pabna R.; Rakietenvirinae; Rosenblumvirus; Rosenblumvirus pabna
Staphylococcus phage TSP R.; Rakietenvirinae; Rosenblumvirus; Rosenblumvirus SCH1
Staphylococcus phage S13 R.; Rakietenvirinae; Rosenblumvirus; Rosenblumvirus S241
Staphylococcus phage vB_SauP-436A1 R.; Rakietenvirinae; Rosenblumvirus; Rosenblumvirus SCH1
Staphylococcus phage SCH111 R.; Rakietenvirinae; Rosenblumvirus; Rosenblumvirus SCH1
Staphylococcus phage SCH1 R.; Rakietenvirinae; Rosenblumvirus; Rosenblumvirus SCH1
Staphylococcus phage vB_Sa_2868B2 R.; Rakietenvirinae; Rosenblumvirus
Staphylococcus phage LSA2366 R.; Rakietenvirinae; Rosenblumvirus; Rosenblumvirus LSA2366
Staphylococcus phage CSA13 R.; Rakietenvirinae; Rosenblumvirus; Rosenblumvirus CSA13
Staphylococcus phage vB_SauP_JS26 R.; Rakietenvirinae; Rosenblumvirus
Staphylococcus phage SAP-2 R.; Rakietenvirinae; Rosenblumvirus; Rosenblumvirus SAP2
Staphylococcus phage SA46-CL1 R.; Rakietenvirinae; Rosenblumvirus; Rosenblumvirus SA46CL1
Staphylococcus phage SA46-CTH2 R.; Rakietenvirinae; Rosenblumvirus
Staphylococcus phage SA44-CTH7 R.; Rakietenvirinae; Rosenblumvirus
Staphylococcus phage SA1-CTA1 R.; Rakietenvirinae; Rosenblumvirus
Staphylococcus phage BP39 R.; Rakietenvirinae; Rosenblumvirus; Rosenblumvirus BP39
Staphylococcus phage SA30-CTH2 R.; Rakietenvirinae; Rosenblumvirus
Staphylococcus phage SA46-CTH4 R.; Rakietenvirinae; Rosenblumvirus
Staphylococcus phage SA03-CTH2 R.; Rakietenvirinae; Rosenblumvirus
Staphylococcus phage 351Saur083PP R.; Rakietenvirinae; Rosenblumvirus
Staphylococcus phage GRCS R.; Rakietenvirinae; Rosenblumvirus; Rosenblumvirus GRCS
Staphylococcus phage vB_SauR_SW21 R.; Rakietenvirinae; Rosenblumvirus
Staphylococcus phage Simurgh R.; Rakietenvirinae; Rosenblumvirus
Staphylococcus phage Huma R.; Rakietenvirinae; Rosenblumvirus
Staphylococcus phage Portland R.; Rakietenvirinae; Rosenblumvirus; Rosenblumvirus EBHT
Staphylococcus phage PSa3 R.; Rakietenvirinae; Rosenblumvirus; Rosenblumvirus PSa3
Staphylococcus phage vB_SauP_phiAGO1.9 R.; Rakietenvirinae; Rosenblumvirus; Rosenblumvirus AGO13
Staphylococcus phage vB_SauP_phiAGO1.3 R.; Rakietenvirinae; Rosenblumvirus; Rosenblumvirus AGO13
Staphylococcus phage 66 R.; Rakietenvirinae; Rosenblumvirus; Rosenblumvirus rv66
Staphylococcus phage phi44AHJD R.; Rakietenvirinae; Rosenblumvirus; Rosenblumvirus rv44AHJD
Staphylococcus phage vB_SauP-V4SA02 R.; Rakietenvirinae; Rosenblumvirus
Staphylococcus phage P68 R.; Rakietenvirinae; Rosenblumvirus
Clostridium phage LPCPA6 G.; Hzauvirus LPCPA6
Clostridium phage DCp1 G.; Susfortunavirus
Clostridium phage susfortuna G.; Susfortunavirus; Susfortunavirus susfortuna
Clostridium phage CPD7 G.; Susfortunavirus; Susfortunavirus susfortuna
Clostridium phage CpV1 G.; Denniswatsonvirinae; Capvunavirus; Capvunavirus CpV1
Clostridium phage vB_CpeP_HN02 G.; Denniswatsonvirinae; Gregsiragusavirus
Clostridium phage cpp G.; Denniswatsonvirinae; Gregsiragusavirus
Clostridium phage CPS1 G.; Denniswatsonvirinae; Gregsiragusavirus; Gregsiragusavirus CPS1
Clostridium phage phi24R G.; Denniswatsonvirinae; Gregsiragusavirus; Gregsiragusavirus phi24R
Clostridium phage vB_CpeP_PM11 G.; Denniswatsonvirinae; Gregsiragusavirus
Clostridium phage CPD2 G.; Denniswatsonvirinae; Gregsiragusavirus; Gregsiragusavirus CPD2
Clostridium phage phiCP7R G.; Brucesealvirus; Brucesealvirus CP7R
Clostridium phage CPQ1 G.; Brucesealvirus; Brucesealvirus CP7R
Clostridium phage CPQ8 G.; Brucesealvirus
Clostridium phage CPQ9 G.; Brucesealvirus
Clostridium phage CPQ10 G.; Brucesealvirus
Clostridium phage CPQ7 G.; Brucesealvirus
Clostridium phage phiCPV4 G.; Brucesealvirus; Brucesealvirus CPV4
Clostridium phage CPQ3 G.; Brucesealvirus
Clostridium phage phiZP2 G.; Brucesealvirus; Brucesealvirus ZP2
Clostridium phage CPS2 G.; Brucesealvirus; Brucesealvirus CPS2
Actinomyces phage Av-1 Dybvigvirus; Dybvigvirus Av1
Bifidobacterium phage BadAargau2 Badaztecvirus; Badaztecvirus badaargau2
Bifidobacterium phage BadAztec1 Badaztecvirus; Badaztecvirus badaztec1
Bifidobacterium phage BadAztec4 Badaztecvirus
Bifidobacterium phage BadAztec2 Badaztecvirus; Badaztecvirus badaztec1
Arthrobacter phage Mendel Anjalivirus; Anjalivirus mendel
Arthrobacter phage Anjali Anjalivirus; Anjalivirus anjali
Curtobacterium phage Pize "Aykavirus"-like
Curtobacterium phage Reje "Aykavirus"-like
Curtobacterium phage Ayka "Aykavirus"
Lactococcus phage asccphi28 S.
Streptococcus phage Cp1 Madridviridae; Cepunavirus; Cepunavirus Cp1
Streptococcus phage SOCP Madridviridae; Cepunavirus; Cepunavirus Cp1
Streptococcus phage CP-7 Madridviridae; Cepunavirus; Cepunavirus Cp7
Amedibacillus phage AD70P3 G.
Amedibacillus phage AD70P2 G.
Acinetobacter phage Phanie Astrithrvirus
Pectobacterium phage DU_PP_III Astrithrvirus
Salmonella phage Astrid Astrithrvirus; Astrithrvirus astrithr
Salmonella phage astrithr Astrithrvirus; Astrithrvirus astrithr
Salmonella phage assan Astrithrvirus; Astrithrvirus astrithr
Bacillus phage Harambe S.; Harambevirus; Harambevirus harambe
Bacillus phage BeachBum S.; Harambevirus; Harambevirus beachbum
Bacillus phage MG-B1 S.; Northropvirinae; Klosterneuburgvirus; Klosterneuburgvirus MGB1
Bacillus phage DLc1 S.; Huangshavirus; Huangshavirus dlcuna
Bacillus phage BC-5 S.; Northropvirinae; Hemphillvirus
Bacillus phage DK1 S.; Northropvirinae; Hemphillvirus; Hemphillvirus DK1
Bacillus phage DK3 S.; Northropvirinae; Hemphillvirus; Hemphillvirus DK3
Bacillus phage DK2 S.; Northropvirinae; Hemphillvirus; Hemphillvirus DK2
Bacillus phage DLn1 S.; Northropvirinae; Hemphillvirus
Bacillus phage vB_BceP_LY3 S.; Northropvirinae
Bacillus phage Aurora S.; Northropvirinae; Claudivirus; Claudivirus aurora
Bacillus phage Claudi S.; Northropvirinae; Claudivirus; Claudivirus claudi
Bacillus phage SerPounce S.; Northropvirinae; Claudivirus; Claudivirus serpounce
Bacillus phage Baseball field S.; Northropvirinae; Claudivirus; Claudivirus baseballfield
Bacillus phage Thornton S.; Northropvirinae; Claudivirus; Claudivirus thornton
Bacillus phage VioletteMad S.; Northropvirinae; Claudivirus; Claudivirus konjotrouble
Bacillus phage KonjoTrouble S.; Northropvirinae; Claudivirus; Claudivirus konjotrouble
Bacillus phage QCM11 S.; Northropvirinae; Claudivirus; Claudivirus QCM11
Bacillus phage vB_BthP-Goe4 S.; Northropvirinae; Claudivirus; Claudivirus Goe4
Bacillus phage Juan S.; Northropvirinae; Claudivirus; Claudivirus juan
Bacillus phage Ademby S.; Northropvirinae; Claudivirus; Claudivirus stitch
Bacillus phage StevenHerd11 S.; Northropvirinae; Claudivirus; Claudivirus stitch
Bacillus phage Stitch S.; Northropvirinae; Claudivirus; Claudivirus stitch
Bacillus phage RadRaab S.; Northropvirinae; Claudivirus; Claudivirus stitch
Bacillus phage VMY22 S.; Mingyongvirus; Mingyongvirus VMY22
Enterococcus phage Athos S.
Bacillus phage vB_Bpu_PumA1 S.; Bundooravirus; Bundooravirus PumA1
Bacillus phage WhyPhy S.; Bundooravirus; Bundooravirus whyphy
Bacillus phage vB_Bpu_PumA2 S.; Bundooravirus; Bundooravirus PumA2
Bacillus phage Karezi S.; Tatarstanvirinae; Karezivirus; Karezivirus karezi
Bacillus phage SRT01hs S.; Tatarstanvirinae; Gaunavirus; Gaunavirus SRT01hs
Bacillus phage vB_BaeroP_SYYB1 S.; Tatarstanvirinae
Bacillus phage GA1 S.; Tatarstanvirinae; Gaunavirus; Gaunavirus GA1
Cytobacillus phage Bfsp1 S.
Bacillus phage Chedec 11 S.; Picovirinae
Bacillus phage B103 S.; Picovirinae; Beecentumtrevirus; Beecentumtrevirus B103
Bacillus phage BSP11 S.; Picovirinae; Beecentumtrevirus
Bacillus phage vB_BsuP-Goe1 S.; Picovirinae; Beecentumtrevirus; Beecentumtrevirus Goe1
Bacillus phage Nf S.; Picovirinae; Beecentumtrevirus; Beecentumtrevirus Nf
Bacillus phage phi29 S.; Picovirinae; Salasvirus; Salasvirus phi29
Bacillus phage vB_BsuP-Goe22 S.; Picovirinae; Salasvirus
Bacillus phage TBA3 S.; Picovirinae; Salasvirus
Bacillus phage vB_BsuP-Goe18 S.; Picovirinae; Salasvirus
Bacillus phage Gxv1 S.; Picovirinae; Salasvirus; Salasvirus Gxv1
Bacillus phage PZA S.; Picovirinae; Salasvirus; Salasvirus PZA
Bacillus phage Whiting18 S.; Picovirinae; Salasvirus; Salasvirus PZA
Bacillus phage Arbo1 S.; Picovirinae; Salasvirus; Salasvirus PZA
Bacillus phage vB_BveP-Goe6 S.; Picovirinae; Salasvirus; Salasvirus Goe6
Bacillus phage vB_BsuP-Goe23 S.; Picovirinae; Salasvirus
Bacillus phage vB_BsuP-Goe15 S.; Picovirinae; Salasvirus
Bacillus phage BSTP6 S.; Picovirinae; Salasvirus; Salasvirus phi29
Bacillus phage BSP4 S.; Picovirinae; Salasvirus
Bacillus phage BSTP4 S.; Picovirinae; Salasvirus; Salasvirus phi29
Bacillus phage BSP2 S.; Picovirinae; Salasvirus
Anjalivirus
Astrithrvirus
Badaztecvirus
Dybvigvirus
Guelinviridae
Madridviridae
Rountreeviridae
Salasmaviridae
Tree scale: 1

Figure S5. Maximum likelihood phylogenetic trees based on amino acid sequences of MCP predicted to be encoded in genomes of isolated φ29-like and related phages. Taxonomy, obtained from the ICTV classification and based on the results of this phylogenetic analysis, is indicated in legends. Branches with a bootstrap support lower than 50% have been deleted. Bootstrap values are shown near their branches. The scale bar shows one estimated substitution per site and the tree was rooted to the midpoint.

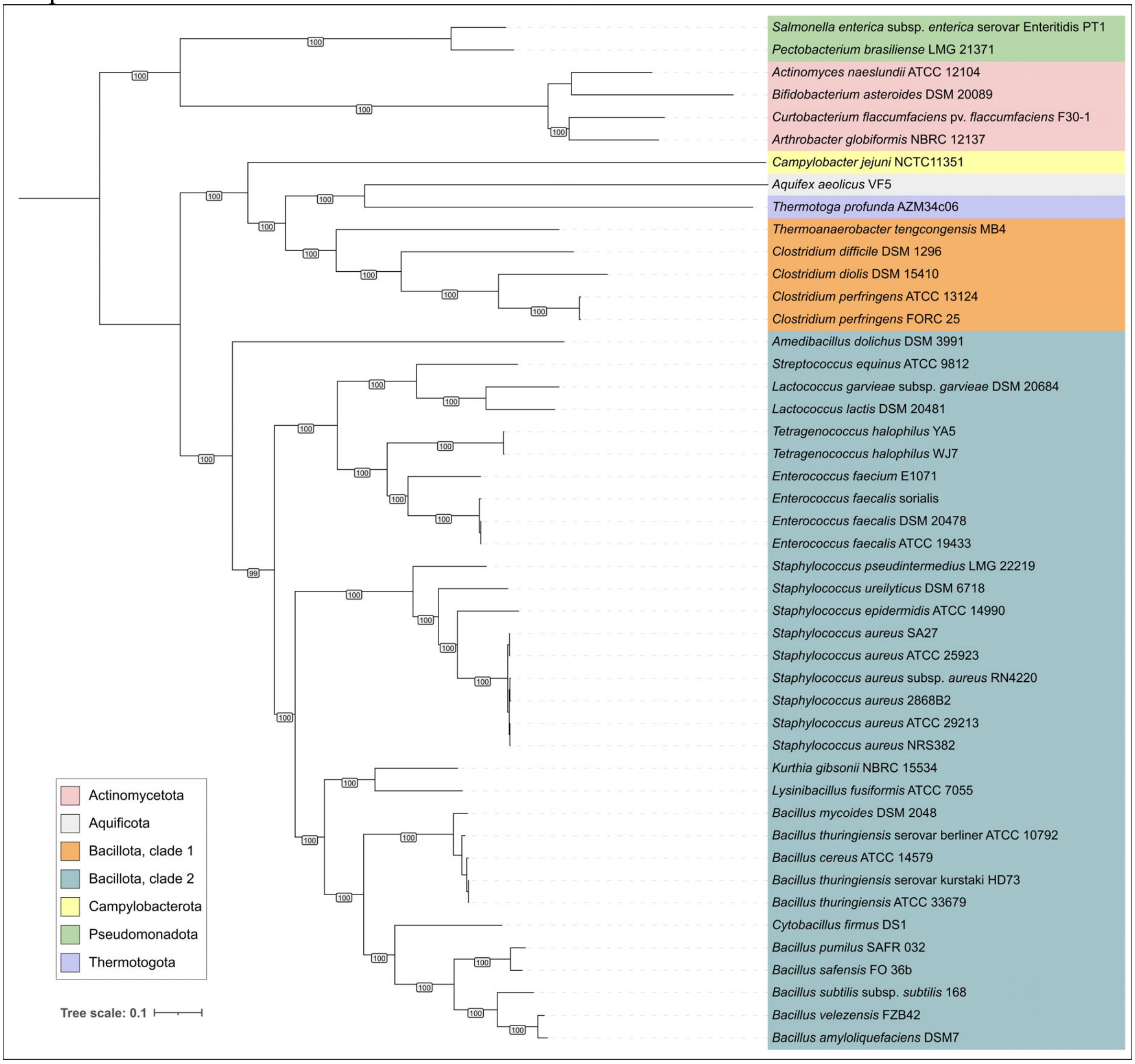

Figure S6. Maximum likelihood phylogenetic tree based on concatenated alignments of 81 bacterial core genes [143], extracted from the genomic sequences of bacterial hosts of φ29-like and related phages, as well as other related bacteria. Bacterial taxonomy is indicated in the legend. Branches with a bootstrap support lower than 50% have been deleted. Bootstrap values are shown near their branches. The scale bar shows 0.1 estimated substitutions per site and the tree was rooted to the midpoint.

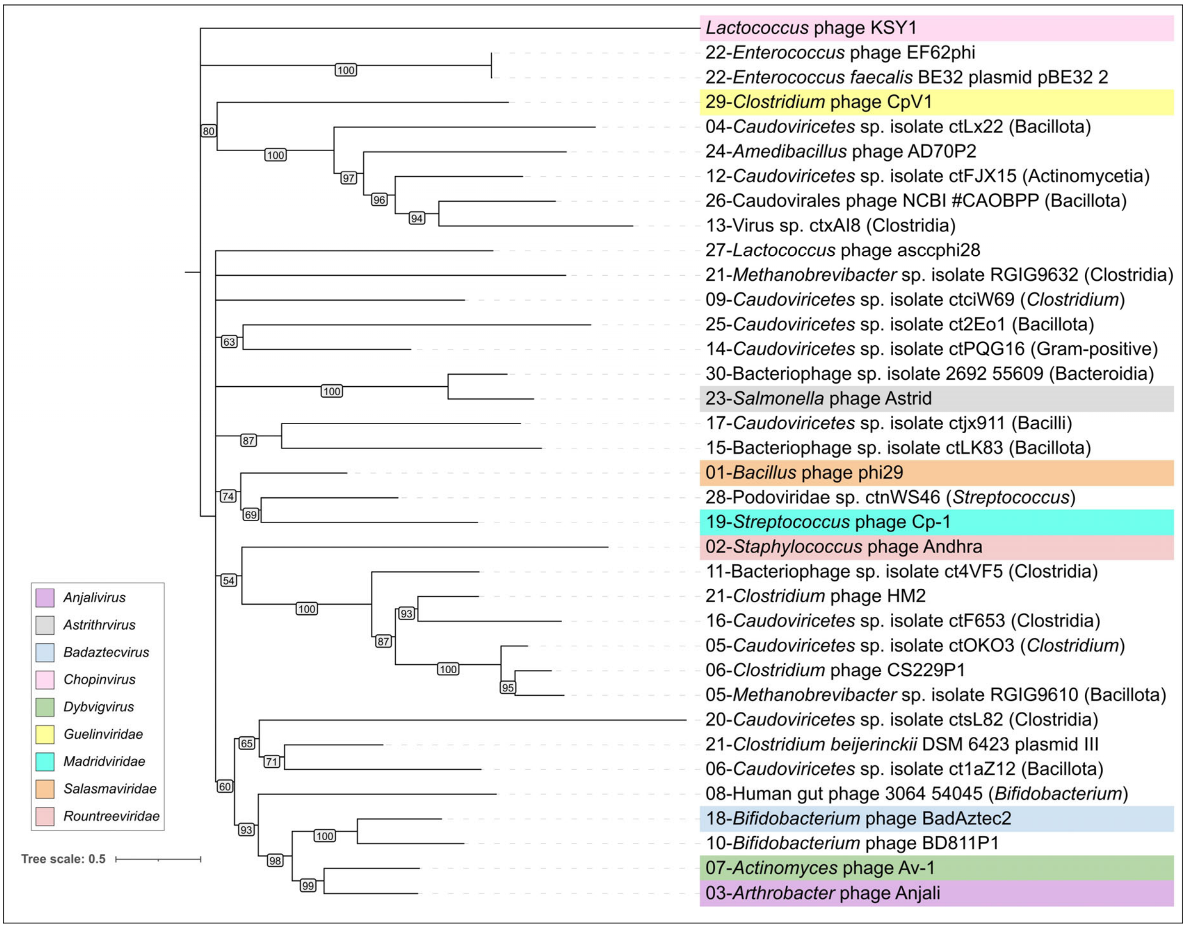
Lactococcus phage KSY1
22-Enterococcus phage EF62phi
22-Enterococcus faecalis BE32 plasmid pBE32 2
29-Clostridium phage CpV1
04-Caudoviricetes sp. isolate ctLx22 (Bacillota)
24-Amedibacillus phage AD70P2
12-Caudoviricetes sp. isolate ctFJX15 (Actinomycetia)
26-Caudovirales phage NCBI #CAOBPP (Bacillota)
13-Virus sp. ctxAI8 (Clostridia)
27-Lactococcus phage asccphi28
21-Methanobrevibacter sp. isolate RGIG9632 (Clostridia)
09-Caudoviricetes sp. isolate ctciW69 (Clostridium)
25-Caudoviricetes sp. isolate ct2Eo1 (Bacillota)
14-Caudoviricetes sp. isolate ctPQG16 (Gram-positive)
30-Bacteriophage sp. isolate 2692 55609 (Bacteroidia)
23-Salmonella phage Astrid
17-Caudoviricetes sp. isolate ctjx911 (Bacilli)
15-Bacteriophage sp. isolate ctLK83 (Bacillota)
01-Bacillus phage phi29
28-Podoviridae sp. ctnWS46 (Streptococcus)
19-Streptococcus phage Cp-1
02-Staphylococcus phage Andhra
11-Bacteriophage sp. isolate ct4VF5 (Clostridia)
21-Clostridium phage HM2
16-Caudoviricetes sp. isolate ctF653 (Clostridia)
05-Caudoviricetes sp. isolate ctOKO3 (Clostridium)
06-Clostridium phage CS229P1
05-Methanobrevibacter sp. isolate RGIG9610 (Bacillota)
20-Caudoviricetes sp. isolate ctsL82 (Clostridia)
21-Clostridium beijerinckii DSM 6423 plasmid III
06-Caudoviricetes sp. isolate ct1aZ12 (Bacillota)
08-Human gut phage 3064 54045 (Bifidobacterium)
18-Bifidobacterium phage BadAztec2
10-Bifidobacterium phage BD811P1
07-Actinomyces phage Av-1
03-Arthrobacter phage Anjali
Anjalivirus
Astrithrvirus
Badaztecvirus
Chopinvirus
Dybvigvirus
Guelinviridae
Madridviridae
Salasmaviridae
Rountreeviridae
Tree scale: 0.5

(**a**)

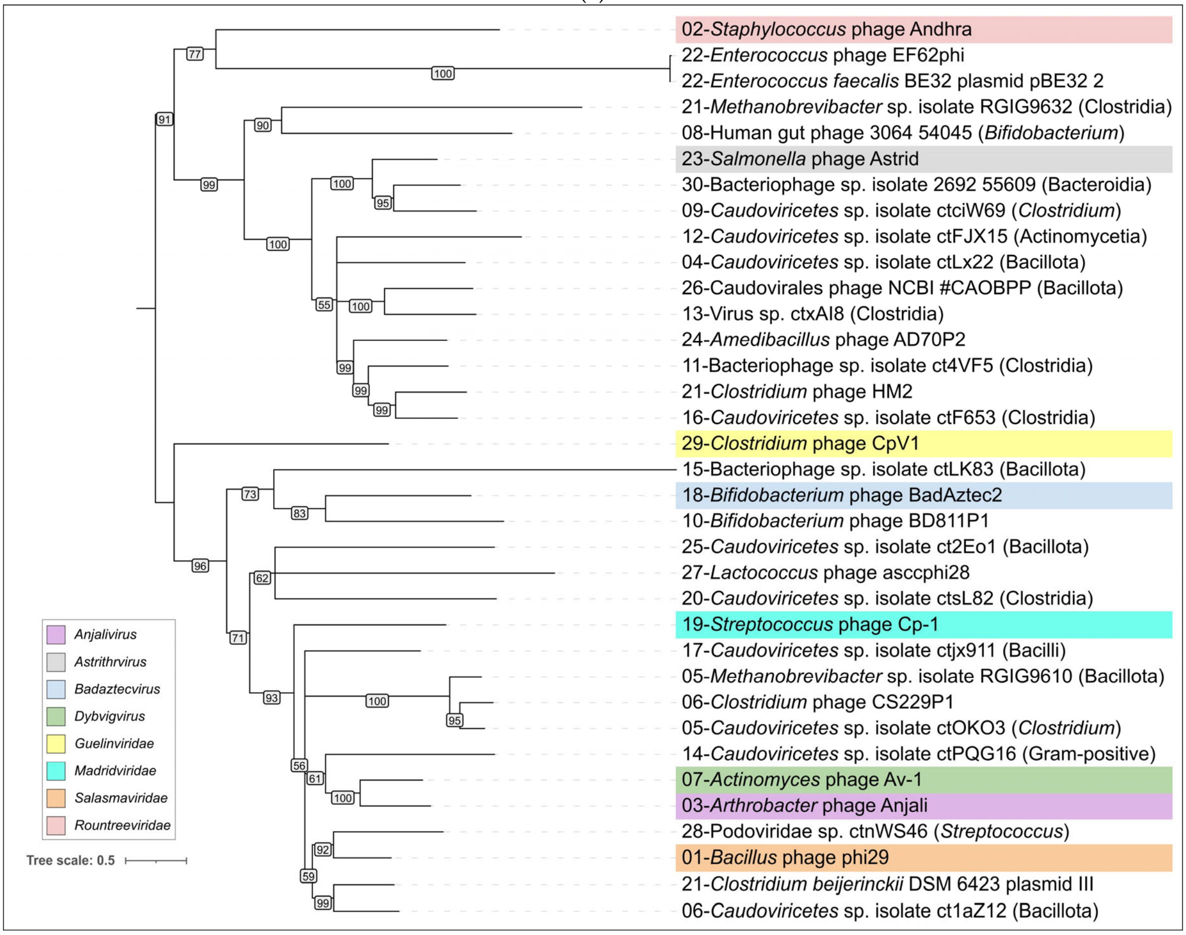
02-Staphylococcus phage Andhra
22-Enterococcus phage EF62phi
22-Enterococcus faecalis BE32 plasmid pBE32 2
21-Methanobrevibacter sp. isolate RGIG9632 (Clostridia)
08-Human gut phage 3064 54045 (Bifidobacterium)
23-Salmonella phage Astrid
30-Bacteriophage sp. isolate 2692 55609 (Bacteroidia)
09-Caudoviricetes sp. isolate ctciW69 (Clostridium)
12-Caudoviricetes sp. isolate ctFJX15 (Actinomycetia)
04-Caudoviricetes sp. isolate ctLx22 (Bacillota)
26-Caudovirales phage NCBI #CAOBPP (Bacillota)
13-Virus sp. ctxAI8 (Clostridia)
24-Amedibacillus phage AD70P2
11-Bacteriophage sp. isolate ct4VF5 (Clostridia)
21-Clostridium phage HM2
16-Caudoviricetes sp. isolate ctF653 (Clostridia)
29-Clostridium phage CpV1
15-Bacteriophage sp. isolate ctLK83 (Bacillota)
18-Bifidobacterium phage BadAztec2
10-Bifidobacterium phage BD811P1
25-Caudoviricetes sp. isolate ct2Eo1 (Bacillota)
27-Lactococcus phage asccphi28
20-Caudoviricetes sp. isolate ctsL82 (Clostridia)
19-Streptococcus phage Cp-1
17-Caudoviricetes sp. isolate ctjx911 (Bacilli)
05-Methanobrevibacter sp. isolate RGIG9610 (Bacillota)
06-Clostridium phage CS229P1
05-Caudoviricetes sp. isolate ctOKO3 (Clostridium)
14-Caudoviricetes sp. isolate ctPQG16 (Gram-positive)
07-Actinomyces phage Av-1
03-Arthrobacter phage Anjali
28-Podoviridae sp. ctnWS46 (Streptococcus)
01-Bacillus phage phi29
21-Clostridium beijerinckii DSM 6423 plasmid III
06-Caudoviricetes sp. isolate ct1aZ12 (Bacillota)
Anjalivirus
Astrithrvirus
Badaztecvirus
Chopinvirus
Dybvigvirus
Guelinviridae
Madridviridae
Salasmaviridae
Rountreeviridae
Tree scale: 0.5

(**b**)

Figure S7. Maximum likelihood phylogenetic trees based on amino acid sequences of Ter (a) and DNAP (b), predicted in sequences representing clusters of φ29-like and related phages. The taxonomy of isolated phages is indicated in legends. Branches with a bootstrap sup-port lower than 50% have been deleted. Bootstrap values are shown near their branches. The scale bar shows 0.5 estimated substitutions per site and the trees were rooted to the midpoint.

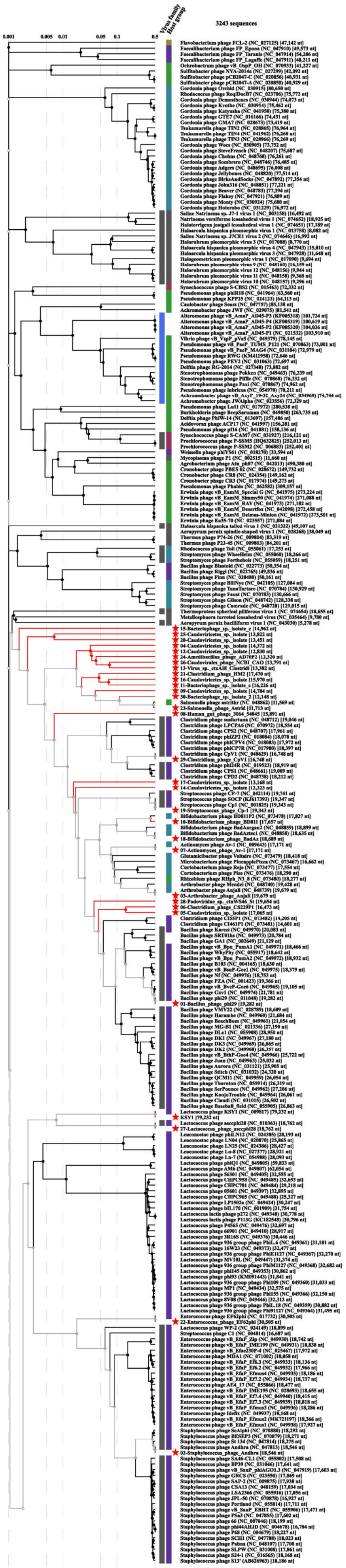
3243 sequences
Virus family
Host group
0.001
0.005
0.01
0.05
0.1
0.5

Figure S8. Proteomic tree of phages representing clusters of φ29-related phages and *Lactococus* phage KSY1 constructed using ViPTree.

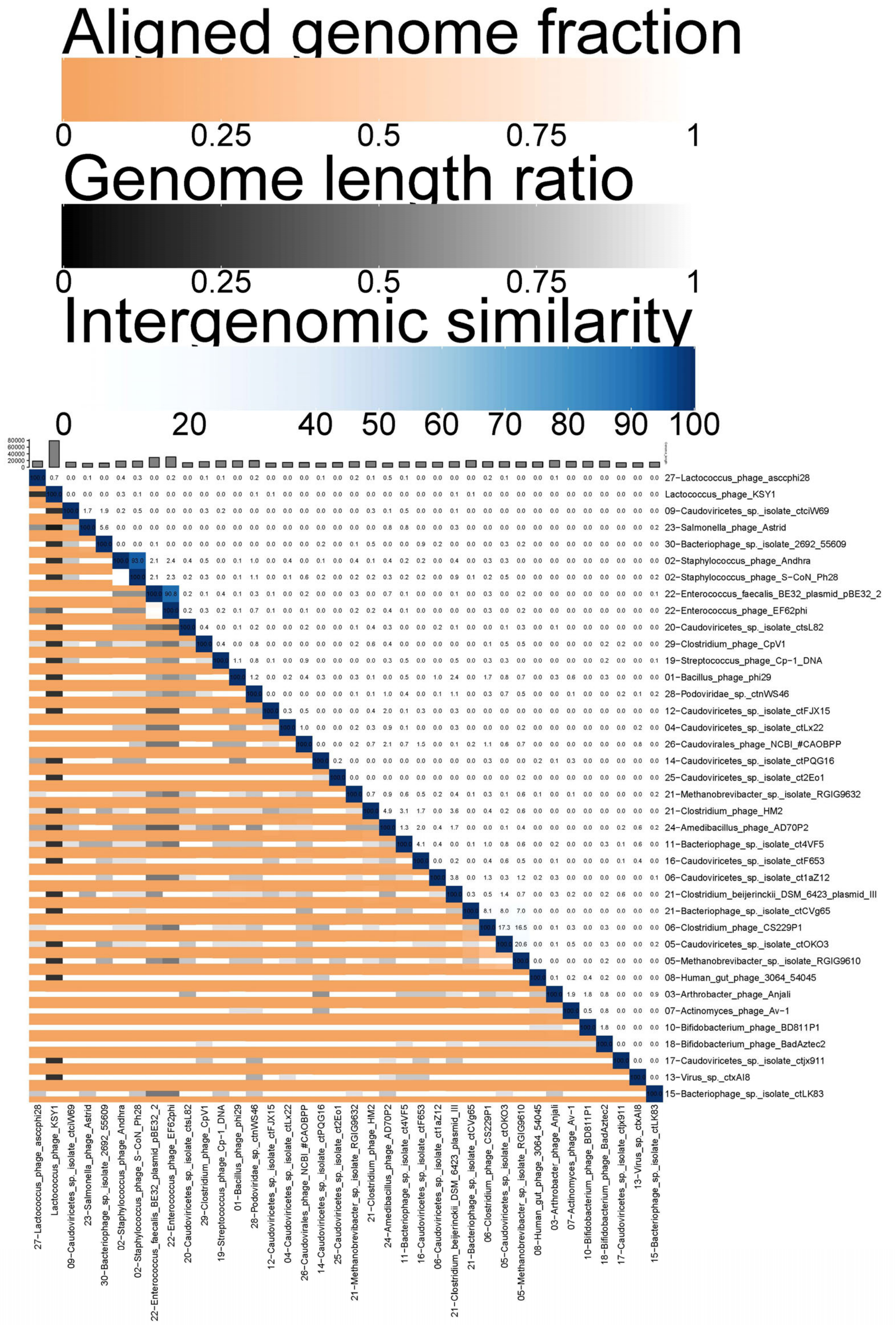

Figure S9. VIRIDIC-generated heatmap of phages representing clusters of φ29-related phages and *Lactococus* phage KSY1. The heatmap incorporates intergenomic similarity values (right half) and alignment indicators (left half and top annotation). In the right half, the colour coding indicated the similarity values according to legend. The numbers represent the similarity values for each genome pair, rounded to the first decimal. In the left half, three indicator values are represented for each genome pair, indicating aligned fraction genome (for the genome found in this row), genome length ratio (for the two genomes in this pair) and aligned fraction genome 2 (for the genome found in this column) according to the legend
